# Lipogenesis-driven EGFR palmitoylation enables metastatic immune evasion in triple-negative breast cancer

**DOI:** 10.64898/2026.05.21.726063

**Published:** 2026-05-26

**Authors:** Myung Soo Ko, Divya Ramchandani, Glenn Simmons, Jason McCormick, Sebastian Carrasco, Arshdeep Singh, Liron Yoffe, Guoan Zhang, Vivek Mittal

**Affiliations:** 1 Department of Cardiothoracic Surgery, Weill Cornell Medicine, New York, NY, USA.; 2 Neuberger Berman Lung Cancer Center, Weill Cornell Medicine, New York, NY, USA.; 3 Sandra and Edward Meyer Cancer Center, Weill Cornell Medicine, New York, NY, USA; 4 Department of Cell and Developmental Biology, Weill Cornell Medicine, New York, NY, USA.; 5 Department of Biomedical Sciences, College of Veterinary Medicine, Cornell University, Ithaca, USA; 6 Flow Cytometry Core Facility, Weill Cornell Medicine, New York, NY, USA; 7 Laboratory of Comparative Pathology, Memorial Sloan Kettering Cancer Center, Weill Cornell Medicine, Rockefeller University, New York, NY, USA; 8 Proteomics and Metabolomics Core Facility, Weill Cornell Medicine, New York, NY, USA.; 9 Caryl and Israel Englander Institute for Precision Medicine, New York, NY, USA

**Keywords:** Triple-negative breast cancer, KRAS-mutant epithelial tumors, Fatty acid synthase (FASN), lung metastasis, metastatic immune escape, Fatty acid metabolism, EGFR palmitoylation, Immunometabolism, PI3K-mTOR signaling, antigen presentation, CD8+ T cells, immunosuppression

## Abstract

Metastasis is the leading cause of death in triple-negative breast cancer (TNBC), yet how tumor cells evade immune recognition during dissemination remain unclear. Here, we show that de novo lipogenesis drives metastatic immune evasion through FASN-mediated palmitoylation of EGFR. This modification establishes a lipid-dependent membrane signaling scaffold that sustains PI3K-AKT-mTOR signaling independently of MAPK compensation, thereby suppressing MHC-I antigen presentation through post-translational regulation. As a result, metastatic-competent cells escape CD8+ T cell surveillance and colonize distant organs. Genetic or pharmacologic inhibition of fatty acid synthase (FASN), or expression of palmitoylation-deficient EGFR mutants restores MHC-I surface expression, unleashes robust CD8^+^ T cell activation, and markedly impairs lung metastasis without affecting primary tumor growth. This lipid-dependent EGFR-PI3K-mTOR axis operates independently of MAPK signaling and is not rescued by exogenous lipids, revealing a non-redundant metabolic dependency. Our results identify FASN-driven EGFR palmitoylation as a tumor-intrinsic immunometabolic checkpoint that couples lipid metabolism to antigen-presentation and metastatic competence. Targeting this pathway with clinically advancing FASN inhibitors offers a promising strategy to suppress metastasis by restoring anti-tumor immunity in TNBC.

## Introduction.

Triple-negative breast cancer (TNBC) is an aggressive disease marked by rapid metastatic spread, and poor responses to immunotherapy^1–3^. Although metastatic dissemination is the leading cause of death, the tumor-intrinsic mechanisms that allow cancer cells to evade immune recognition during metastatic progression remain poorly understood. Emerging evidence implicates de novo lipogenesis in tumor progression and immune regulation. Fatty acid synthase (FASN), the rate-limiting enzyme in de novo fatty acid synthesis, is frequently upregulated in aggressive cancers and correlates with poor patient outcomes^4^. Beyond supporting membrane biogenesis, FASN produces palmitate that can modify signaling proteins via palmitoylation. These observations suggests that de novo lipogenesis sustains immune-evasive signaling states during metastatic progression. However, whether lipogenesis directly regulates antigen presentation during metastasis remains unknown.

Here we identify a lipid-dependent signaling axis in which FASN-derived palmitate promotes EGFR palmitoylation, thereby sustaining downstream PI3K-AKT-mTOR signaling and suppressing MHC class I antigen presentation. These findings reveal a non-canonical role for EGFR in metastatic progression and identify a therapeutically actionable link between de novo lipid synthesis and metastatic immune escape in TNBC.

### Lipogenic signaling drives metastatic progression in TNBC.

TNBC cells rely heavily on de novo lipogenesis to support proliferation, migration, and survival^5^. However, the mechanisms linking this metabolic program to metastatic progression and immune evasion have remained unclear.

To directly address this, we used a SOX2/OCT4-GFP promoter reporter system^6^ that prospectively identifies a highly metastatic subpopulation in TNBC. In both human MDA-MB-231 cells and murine E0771-ML1 TNBC models^7–9^, these GFP^+^ cells express high levels of stemness transcription factors (SOX2, OCT4 and NANOG), show increased chromosomal instability (CIN)^10^, and exhibit markedly superior metastatic capacity in vivo.

**Extended Data Fig. 1|:**
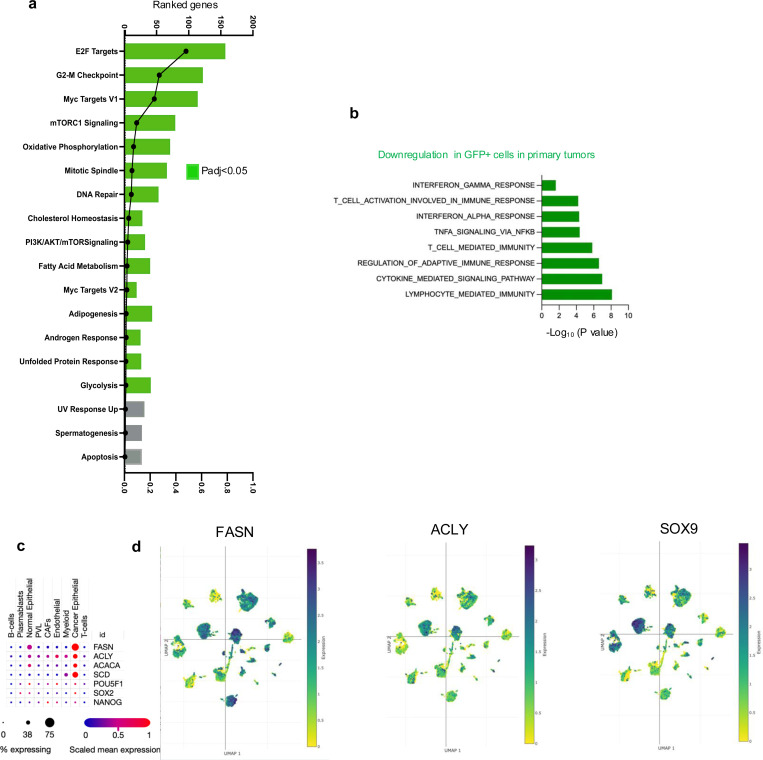
FASN is predominantly expressed in human breast cancer. **a-b,** GSEA of GFP+ versus GFP- tumor cells isolated from orthotopic E0771 primary tumors in vivo (n = 5 biological replicates per group), showing pathways (**a**), enriched (**b**) downregulated in GFP+ metastatic cells relative to GFP- non-metastatic cells. **c-d,** scRNA-seq analysis of TNBC patient samples to assess metabolic pathway activity at single-cell resolution. (**c**) Bubble plot of Fatty acid metabolism genes and metastatic markers (% of cells and expression levels) in major cell types in single cell breast cancer atlas. **(d)** UMAP clustering of cancer epithelial cells showing expression of FASN, ACLY in Sox9 stem cell enriched subpopulations.

**Fig. 1 |:**
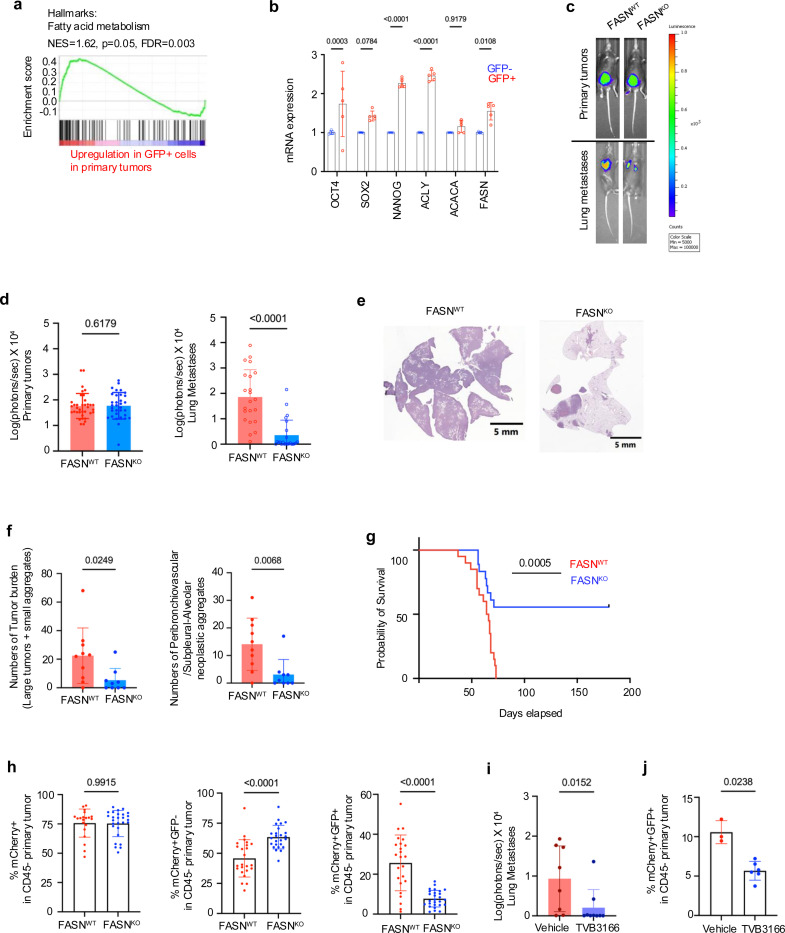
Lipogenic signaling drives metastatic progression in TNBC. **a,** Gene set enrichment analysis (GSEA) of GFP+ compared to GFP- tumor cells isolated from orthotopic EO771 primary tumors (n = 5 biological replicates per group), showing enrichment of fatty acid metabolism in GFP+ cells. **b,** RT-qPCR analysis of lipogenic genes in GFP+ and GFP-tumor cells isolated from orthotopic primary tumors (n=5 biological replicates per group). **c,** Representative bioluminescence imaging (BLI) of primary tumors in vivo at week 4 and lung metastases at week 3 post following primary tumor resection. **d,** Quantification of BLI signals for primary tumors (left panel) and lung metastases (right panel), n= 20 mice. **e**, Representative whole lung histology showing metastatic tumor burden from FASN^WT^ and FASN^KO^ mice at week 3 following primary tumor resection. Scale bars, 5 mm. (**f**). Quantification of total tumor burden per mouse (n= 9), including both large tumors and small neoplastic aggregates (left), and quantification of peribronchiovascular, alveolar, and subpleural neoplastic aggregates (right). **g,** Kaplan-Meier survival curves of FASN^WT^ and FASN^KO^ tumor bearing mice (n=15 per group). **h,** Flow cytometry analysis of mCherry^+^ total tumor cells (left panel), GFP⁻ non-metastatic tumor cells (middle panel), and GFP^+^ (right panel) metastatic tumor cells within orthotopic primary tumors, (n= 20 tumors per group). **i,** Pharmacologic inhibition of lipogenesis using FASN inhibitor TVB-3166 in orthotopic E0771 tumor bearing mice showing quantification of lung metastases (n= 8). **j** Flow cytometry quantification of GFP^+^ metastatic cells following vehicle or TVB-3166 treatment. Data in panels a,b,d,f,h-j are presented as mean ± s.e.m. Statistical significance was determined using two-tailed unpaired Student’s t-tests or a two-way ANOVA for comparisons between two groups. Kaplan-Meier survival curves in panel g were analyzed using the log-rank (Mantel-Cox) test. Exact P values are indicated in the figures. A P value <0.05 was considered statistically significant.

RNA-seq analysis of sorted OCT4/SOX2-GFP^+^ metastatic cells from orthotopic primary tumors revealed selective enrichment of lipogenic and anabolic metabolic pathways, including fatty acid metabolism (Fig. 1a and Extended Data Fig. 1a). qPCR validation confirmed upregulation of key de novo lipogenesis genes, including ATP citrate lyase (ACLY), acetyl-CoA carboxylase 1 (ACC1 encoded by ACACA), and as fatty acid synthase (FASN) (Fig. 1b).

To establish clinical relevance, we analyzed single-cell RNA-seq datasets from TNBC patients^11^. Fatty acid metabolism signatures were significantly enriched in malignant epithelial cells with the highest expression of lipogenic genes observed in subpopulations expressing stemness markers (Extended Data Fig. 1c-d). These data indicate that a distinct lipogenic program is associated with metastatic cell states in both experimental models and human TNBC.

**Extended Data Fig. 2 |:**
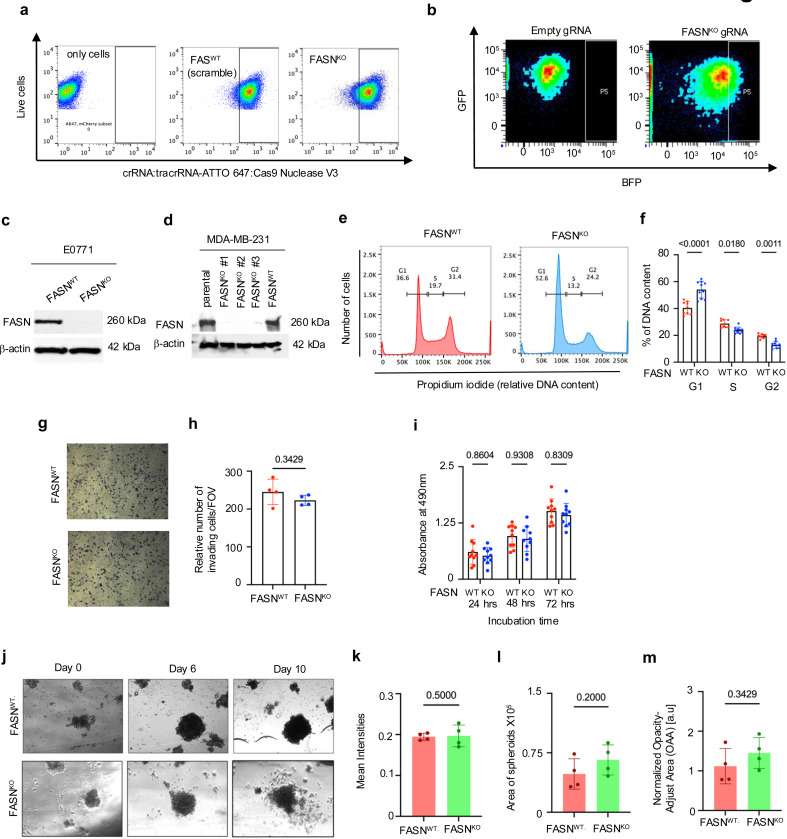
CRISPR-Cas9–mediated FASN knockout in TNBC cells in vitro. **a-d,** A647 or BFP labeled CRISPR-Cas9 mediated knockout of FASN in (**a**), E0771 or (**b**), MDA-MB-231 cells. Non-targeting scramble gRNA cells were used as a negative control. **c-d,** Western blots analysis confirming FASN knockout in (**c**), E0771 or (**d**) MDA-MB-231-LM2 cells. **e-f,** (**e**), Cell cycle analysis (**f**) quantification of biological replicates in FASN^WT^ and FASN^KO^. **g-h.** Invasion assay of FASN^WT^ and FASN^KO^ cells (**g**), microscopy images, (**h**) quantification of biological replicates per group. **i,** Cell proliferation assay of FASN^WT^ and FASN^KO^ . **j–m,** In vitro bioprinting of spheroids derived from FASN^WT^ and FASN^KO^ E0771 cells, cultured in complete media over 0, 6, and 10 days. (n = 4 biological replicates per group).(**j**) Representative images of FASN^WT^ (**top panel**) and FASN^KO^ (**bottom panel**) spheroids over 1, 6, and 10 days. (**k**) Quantification of Mean Intensities at Day 10. (**l**) Quantification of Area of spheroids at Day 10 (**m**) Quantification of Normalized Opacity-Adjust Area (OAA) [a.u] at Day 10. Data in panels f, h-I, k-m are presented as mean ± s.e.m. Statistical significance was determined using two-tailed unpaired Student’s t-tests or a two-way ANOVA for comparisons between two groups. Exact P values are indicated in the figures. A P value <0.05 was considered statistically significant.

**Extended Data Fig. 3 |:**
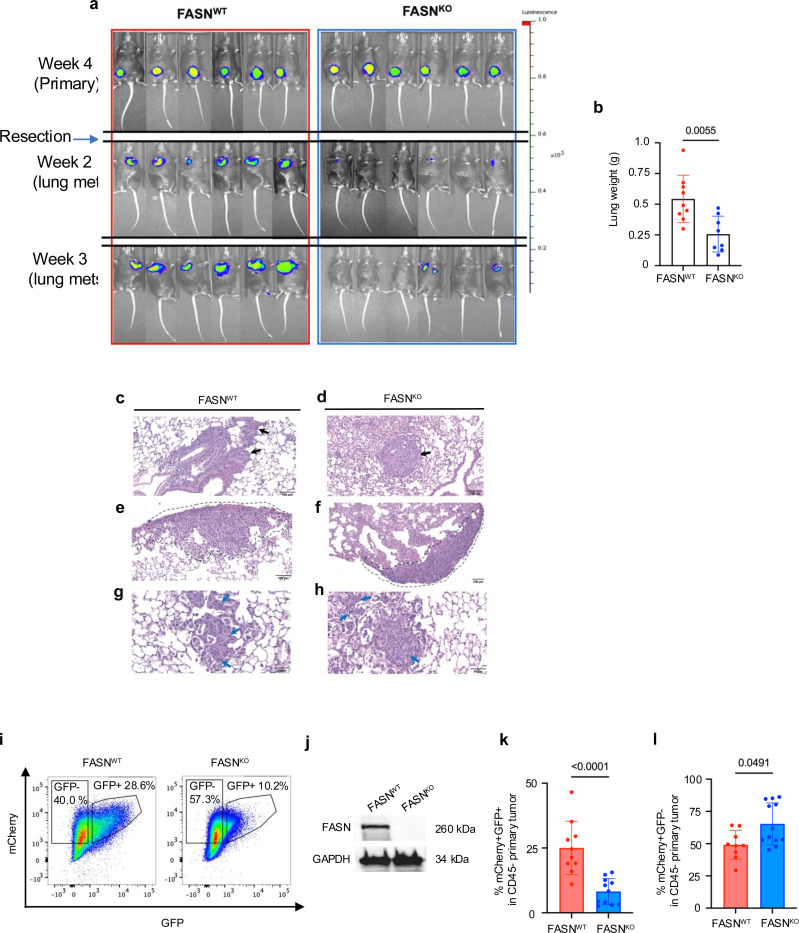
FASN loss reduces TNBC lung metastasis. **a,** Representative bioluminescence imaging (BLI) of primary tumors at week 4 and lung metastases at week 2 and 3 post–primary tumor resection in orthotopic E0771 models (n = 6 biological replicates per group). Tumors were derived from FASN^WT^ and FASN^KO^ E0771 cells. **b,** Quantification of lung metastasis weight (n = 6 biological replicates per group). **c-h,** Representative lung histology from Lung metastases from FASN^WT^ and FASN^KO^ E0771 orthotopic mouse models showing aggregates of neoplastic cells surrounding bronchioles and blood vessels, as well as expanding subpleural spaces and alveoli. (**c**) Peribronchiolar and perivascular spaces are infiltrated by densely packed TNBC cells (black arrows). (**d**) Perivascular space is expanded by a dense aggregate of TNBC cells (black arrows) and is surrounded by hemorrhage and foamy macrophages containing erythrocytes and cellular debris. (**e-f**) Subpleural spaces are expanded by dense aggregates of TNBC cells (dotted lines). (**g-h**) Alveolar septa are expanded by small clusters of TNBC cells (blue arrows) admixed with low numbers of macrophages and erythrocytes. Scale bars, 50 µm and 100 µm. **i,** Representative flow cytometry analysis of GFP+ and GFP- tumor cell populations isolated from orthotopic E0771 primary tumors. **j,** Western blot analysis of FASN expression in FASN^WT^ and FASN^KO^ tumor cells isolated from orthotopic E0771 primary tumors. **k,l,** Quantification of flow cytometry analysis of tumor cell populations from orthotopic E0771 primary tumors generated by transplantation of a 2:1 GFP^+^:GFP⁻ mixture of FASN^WT^ or FASN^KO^ E0771 cells, showing (**k**), GFP+ tumor cells and (**l**) GFP- tumor cells. (n = 10 biological replicates per group). Data in panels b, k-l are presented as mean ± s.e.m. Statistical significance was determined using two-tailed unpaired Student’s t-tests for comparisons between groups. Exact P values are indicated in the figures. A P value <0.05 was considered statistically significant.

To define the role of de novo lipid synthesis in metastatic progression, we focused on FASN, the rate limiting enzyme in palmitate synthesis. FASN is currently being evaluated as a therapeutic target in advanced cancers, including brain metastasis (NCT03179904). We used CRISPR to generate FASN knockout (FASN^KO^) in both murine E0771 and human MDA-MB-231-LM2 TNBC models (Extended Data Fig. 2a-d). FASN deficiency caused only modest changes in cell-cycle distribution and had minimal effects on in vitro cell proliferation, invasion, or spheroid formation in 3D bioprinted matrices (Extended Data Fig. 2e-m). In vivo, FASN loss did not affect orthotopic primary tumor growth in syngeneic immunocompetent mice (Fig.1c-d and Extended Data Fig. 3a), however, it strongly suppressed metastatic outgrowth. After surgical resection of primary tumors (1cm^3^ at week 4), FASN^KO^ tumors showed markedly reduced lung metastasis compared with FASN^WT^ controls, as measured by BLI and lung weight (Fig. 1c-d and Extended Data Fig. 3a-b). Histopathology confirmed a striking reduction in both the number and size of the metastatic lesions (Fig.1e-f and Extended data Fig. 3c-h). This reduced metastatic burden was associated with significantly improved overall survival (Fig. 1g).

To understand how FASN deficiency reduces metastatic burden, we examined the primary tumors. As expected, FASN^KO^ tumors showed clear FASN deficiency (Extended data Fig. 3j) and contained a significantly reduced proportion of OCT4/SOX2-GFP^+^ metastatic cells compared to controls (Fig.1h and Extended data Fig. 3i). These results indicate that de novo lipid synthesis supports the maintenance of metastatic cell states. To test whether this defect could be rescued by increasing the starting number of metastatic cells, we orthotopically injected mixtures of enriched GFP^+^ cells (at a 2:1 GFP^+^ to GFP⁻ ratio). However, enriching for GFP^+^ cells failed to rescue metastatic growth in FASN^KO^ tumors (Extended Data Fig. 3k-l). This confirms that lipogenesis is required for maintenance and progression of metastatic-competent cells.

**Extended Data Fig. 4 |:**
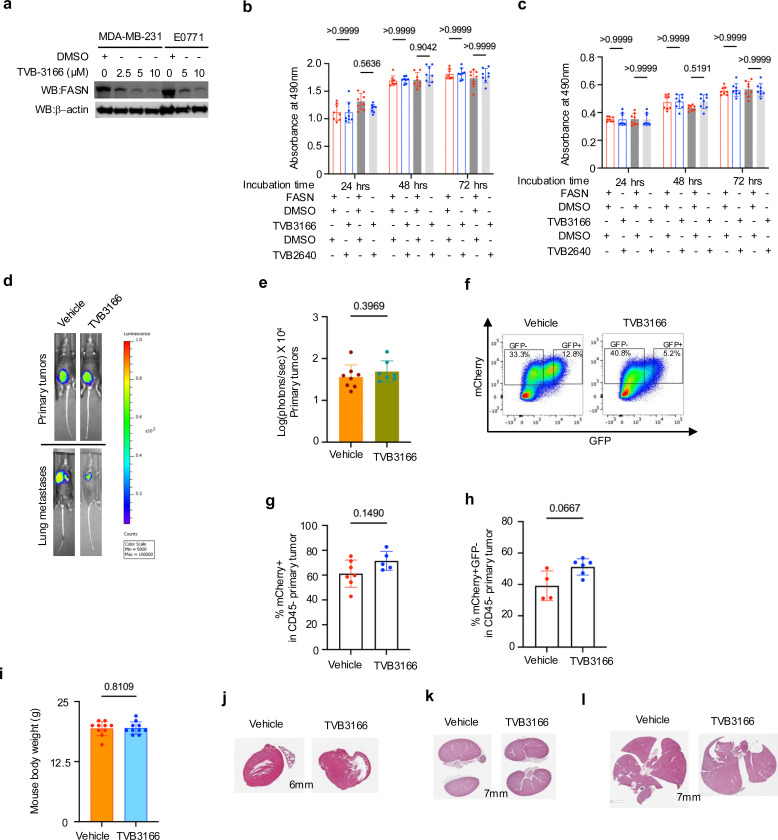
Pharmacologic inhibition of FASN decreases TNBC progression in vivo. **a,** Western blot analysis of FASN expression in MDA-MB-231 and E0771 cells following treatment with DMSO or TVB-3166 (0, 2.5, 5, 10 µM) for 72 h. **b,c**, In vitro cytotoxicity assays assessing cell viability in (**b**), E0771 and (**c**) MDA-MB-231 cells treated with TVB-3166 (10 µM) or TVB-2640 (10 µM) at 0, 24, 48, and 72 h. (n = 8 biological replicates per group). **d,e,** Efficacy of pharmacologic inhibition with vehicle or TVB-3166 in orthotopic FASN^WT^ E0771 in female C57BL/6 mice. (n = 7 biological replicates per group). (**d**) Representative bioluminescence imaging (BLI) of primary tumors and lung metastases. (**e**) Quantification of BLI signal in primary tumors at week4. **f-h,** Representative flow cytometry analysis of tumor cell populations isolated from orthotopic primary tumors treated with vehicle or TVB-3166, showing (**f**), Representative flow cytometry plots of GFP+ and GFP- tumor cell populations (**g**), mCherry+ cells representing whole tumor cells (**h**) GFP- cells. **i–l,** In vivo toxicity assessment following TVB-3166 treatment compared to vehicle, including (**i**), body weight and histological analysis of (**j**), heart, (**k**), kidney, and (**l**) liver. Scale bars, 6 mm (heart) and 7 mm (kidney and liver). Data in panels b-c, g-h are presented as mean ± s.e.m. Statistical significance was determined using a two-way ANOVA or two-tailed unpaired Student’s t-tests for comparisons between two groups. Exact P values are indicated in the figures. A P value <0.05 was considered statistically significant.

Pharmacologic inhibition of FASN produced similar results. We tested two inhibitors: TVB3166 (a preclinical analog), and TVB2640 (a clinically advanced compound currently in phase I clinical trials^4^). Both the inhibitors reduced FASN protein levels in a dose-dependent manner (Extended Data Fig. 4a) without significantly affecting in vitro cell proliferation (Extended Data Fig. 4b-c). In vivo, we began FASN inhibitor treatment one week after tumor implantation. The treatment did not impair primary tumor growth but markedly suppressed lung metastatic burden and reduced the population of metastatic cells (Fig. 1i and Extended Data Fig. 4d-e) (Fig. 1j and Extended Data Fig. 4f-h), and TVB-3166 was well tolerated, with no significant changes in mouse body weight and no detectable organ toxicity (Extended Data Fig. 4i-l).

Together, these genetic and pharmacological data show that blocking de novo lipogenesis selectively impairs metastatic competence while largely sparing primary tumor growth. These findings identify lipogenesis as a critical metabolic dependency that sustains metastatic cell states and progression in TNBC.

### Lipogenic signaling sustains metastatic cell states within primary tumors.

To elucidate how de novo lipogenesis supports metastatic competence, we systematically profiled metabolic dependencies and the downstream signaling pathways across major stages of the metastatic cascade.

**Extended Data Fig. 5 |:**
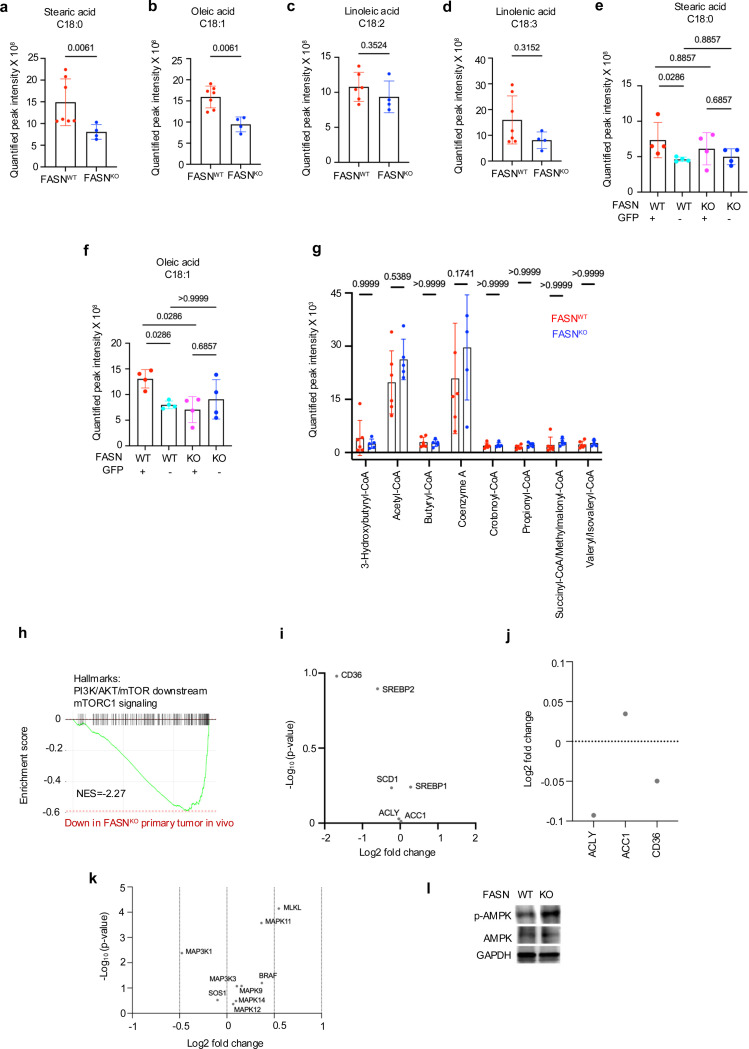
FASN-driven metabolic reprogramming enhances metastasis-initiating cells in primary tumors. **a-f,** LC–MS analysis of free fatty acid abundance, including (**a**) stearic acid, (**b**) oleic acid (**c**) linoleic acid, and (**d**) linolenic acid in FASN^WT^ and FASN^KO^ E0771 primary tumors (n = 6 biological replicates per group). LC–MS analysis of (**e**) stearic acid, (**f**) oleic acid in GFP+ and GFP- cells isolated from orthotopic E0771 primary tumors (n = 4 biological replicates per group). **g,** LC–MS analysis of short-chain acyl-CoAs in FASN^WT^ and FASN^KO^ primary tumors (n = 6 biological replicates per group). **h-k,** Integrated transcriptomic and proteomic analyses of FASN^WT^ compared to FASN^KO^ E0771 primary tumors (n = 5 biological replicates per group). (**h**) GSEA plot of FASN^WT^ compared to FASN^KO^ primary tumors. (**i**), Differentially expressed genes associated with fatty acid synthesis. (**j**), protein expression associated with fatty acid synthesis. (**k**) Transcriptomic analysis of DEGs associated with MAPK signaling pathway. **l,** Western blot analysis of AMPK signaling in FASN^WT^ compared to FASN^KO^ tumor cells isolated from orthotopic E0771 primary tumors. Data in panels a-d, e-f are presented as mean ± s.e.m. Statistical significance was determined using a one-way ANOVA, a two-way ANOVA or two-tailed unpaired Student’s t-tests for comparisons between two groups. Exact P values are indicated in the figures. A P value <0.05 was considered statistically significant.

**Fig. 2 |:**
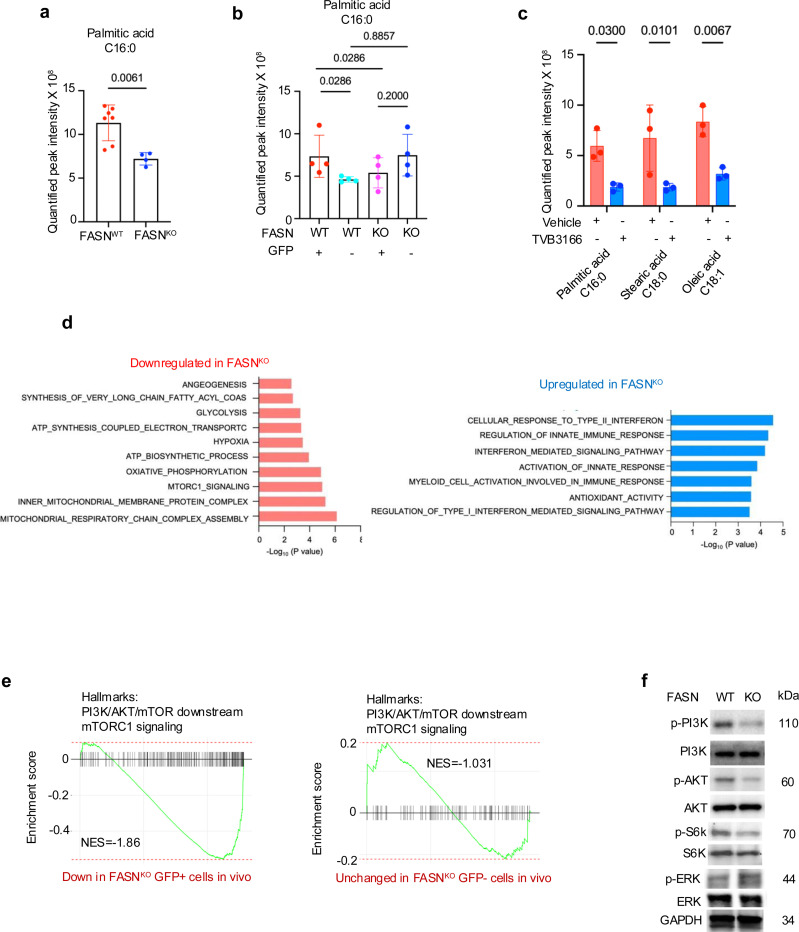
FASN sustains metabolic and signaling states in metastatic cells. **a-b,** LC-MS analysis of palmitic acid abundance in (**a**), FASN^WT^ and FASN^KO^ orthotopic primary tumors and in (**b**), GFP^+^ and GFP⁻ cells isolated from orthotopic EO771 primary tumors (n=5 biological replicates per group). **c,** LC-MS analysis of free fatty acid abundance (palmitic acid, stearic acid, and oleic acid) in E0771 orthotopic primary tumors treated with vehicle or TVB-3166 (n=5 biological replicates per group). **d,** Transcriptomic analysis of FASN^WT^ and FASN^KO^ E0771 orthotopic primary tumors (n = 5 biological replicates per group). (**left panel**) Gene set enrichment analysis (GSEA) in downregulated pathways in FASN^KO^, (**right panel**) upregulated pathways in FASN^KO^. **e**, Gene set enrichment analysis (GSEA) showing pathways in tumor cells isolated from E0771 orthotopic primary tumors (n=5 biological replicates per group). (**left panel**) GSEA plot of FASN^WT^ compared to FASN^KO^ in GFP^+^ cells. (**right panel**) GSEA plot of FASN^WT^ compared to FASN^KO^ in GFP^™^ cells. **f,** Western blot analysis of PI3K-AKT-mTOR and MAPK signaling in FASN^WT^ compared to FASN^KO^ tumor cells isolated from E0771 orthotopic primary tumors. Data in panels a,b,c,d,e are presented as mean ± s.e.m. Statistical significance was determined using two-tailed unpaired Student’s t-tests, a one-way ANOVA or a two-way ANOVA for comparisons between groups. Exact P values are indicated in the figures. A P value <0.05 was considered statistically significant.

Lipidomic analysis revealed that FASN-competent (FASN^WT^) tumors were highly enriched in free fatty acids, especially in palmitic, stearic, and oleic acids (Fig. 2a and Extended Data Fig. 5a-d). Pharmacologic inhibition FASN with TVB3166 produced similar a metabolic shift (Fig. 2c). Importantly, sorted OCT4/SOX2-GFP^+^ metastatic cells from FASN^WT^ tumors displayed markedly higher levels of free fatty acids than GFP^™^ non-metastatic cells. This difference disappeared in FASN-deficient tumors, where GFP^+^ and GFP^™^ cells showed comparable fatty acid levels (Fig. 2b and Extended Data Fig. 5e-f). In contrast, upstream TCA cycle intermediates remained unchanged (Extended Data Fig. 5g). These results establish de novo lipogenesis as a defining metabolic feature of metastatic-competent cells within orthotopic primary tumors.

We next performed transcriptomic profiling to uncover how suppression of lipogenic signaling reprograms pathways linked to metastatic progression. FASN-deficient tumors showed coordinated downregulation of gene sets involved in lipid elongation, mitochondrial bioenergetics, ATP synthesis, OXPHOS, respiratory chain assembly, and hypoxia response (Fig. 2d left panel). At the same time, immune-related gene signatures were upregulated, including pathways for antigen presentation, T cell activation, and interferon responses (Fig. 2d **right panel**). Expression of canonical lipogenic regulators, including SREBP1, SREBP2, and the fatty acid transporter CD36 remained largely unchanged in both transcript and protein levels (Extended Data Fig. 5i-j).

Gene set enrichment analysis (GSEA) showed elevated mTORC1 signaling in FASN^WT^ tumors (Extended Data Fig. 5h). This increase was particularly pronounced in OCT4/SOX2-GFP^+^ metastatic cells and was selectively attenuated upon FASN deletion in this subpopulation, while remaining largely unaffected in GFP⁻ cells (Fig. 2e). Notably, FASN deficiency did not increase MAPK signaling (Extended Data Fig. 5k).

Together, these findings indicate that de novo lipid synthesis supports a broad metabolic and signaling program in metastatic TNBC cells. This program includes enhanced mitochondrial fitness, PI3K-AKT-mTORC1 activity, and immune regulation. It does not rather solely on canonical lipogenic transcriptional programs. Importantly, this lipogenesis-dependent PI3K-mTORC1 signaling occurs independently of compensatory MAPK pathway activation.

### Lipogenic signaling suppresses cell surface MHC-I expression through post-.translational regulation.

Given the upregulation of immune-related gene signatures upon FASN suppression, we next asked whether de novo lipogenesis reshapes the tumor immune microenvironment to promote immune evasion. Metastatic-competent cells, which depend most heavily on lipogenesis and mTORC1 signaling, are especially well positioned to use this pathway during dissemination.

**Extended Data Fig. 6 |:**
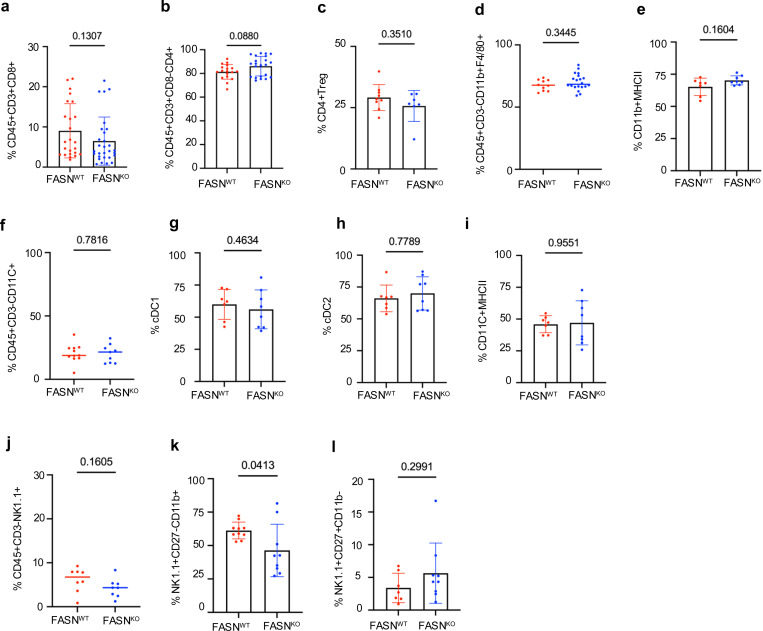
Immune profiling following genetic inhibition of FASN in orthotopic mouse models. **a–l,** Flow cytometry analysis of immune cell populations in FASN^WT^ and FASN^KO^ E0771 primary tumors (n = 8 biological replicates per group), including (**a**), CD8^+^ T cells, (**b**), CD4^+^ T cells, (**c**), regulatory T (T_reg) cells, (**d**), macrophages, (**e**), MHC-II^+^ macrophages, (**f**), dendritic cells (DCs), (**g**), type I DCs, (**h**), type II DCs, (**i**), MHC-II^+^ DCs, (**j**), NK cells, (**k**), mature NK cells, and (**l**) immature NK cells. Data in panels a-l are presented as mean ± s.e.m. Statistical significance was determined using two-tailed unpaired Student’s t-tests for comparisons between two groups. Exact P values are indicated in the figures. A P value <0.05 was considered statistically significant.

**Fig. 3 |:**
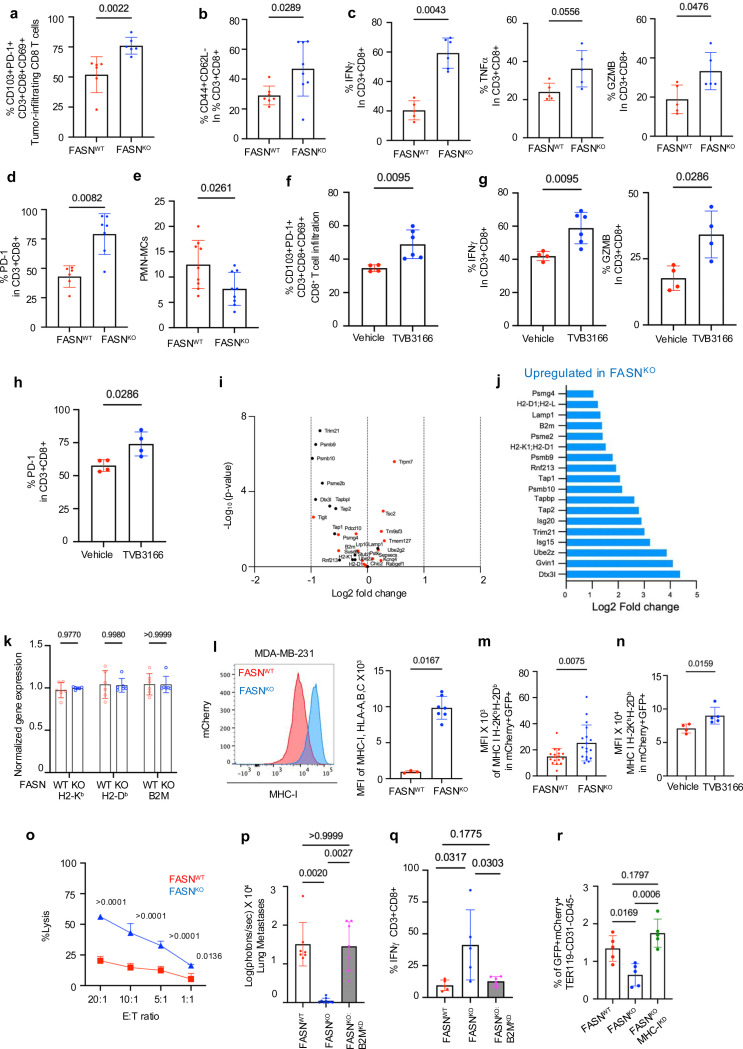
Lipogenic signaling suppresses cell surface MHC-I expression through post-translational regulation. **a-e,** Flow cytometry based immune profiling of FASN^WT^ versus FASN^KO^ E0771 orthotopic primary tumors (n = 6 biological replicates per group), including quantification of (**a**) total CD8^+^ T cell infiltration, (**b**) effector-liker CD8^+^ T cells, (**c**) expressing IFNγ^+^, TNFα^+^, and granzyme B^+^ CD8^+^ T cells. (**d**) PD-1^+^ CD8^+^ T cells. (**e**) polymorphonuclear myeloid-derived suppressor cells (PMN-MDSCs). **f-h,** Flow cytometry based immune profiling from FASN^WT^ E0771 orthotopic primary tumors treated with vehicle or TVB-3166 (n = 6 biological replicates per group), including quantification of (**f**) total CD8^+^ T cell infiltration **(g)** expressing IFNγ^+^ CD8^+^ T cells and granzyme B^+^ CD8^+^ T cells **(h)** PD-1^+^ CD8^+^ T cells. **i,j,** Transcriptomic and proteomic analyses of differentially expressed genes (DEGs) and protein expressions associated with MHC-I antigen processing machinery in FASN^WT^ versus FASN^KO^ E0771 orthotopic primary tumors (n = 5 biological replicates per group), including **(i)**, DEGs (positive regulators, black; negative regulators, red). **(j).** Proteins expressions. **k,** qPCR analysis of mRNA expression of MHC-I subunits in GFP+ cells isolated from FASN^WT^ and FASN^KO^ E0771 orthotopic primary tumors (n = 5 biological replicates per group). **l,** Flow cytometry analysis of cell surface MHC-I in MDA-MB-231 cells (n = 6 biological replicates per group). representative histograms of cell surface MHC-I (**left panel**). Quantification of cell surface MHC-I (**right panel**). **m,n** Flow cytometry analysis of cell surface MHC-I. (**m**), Quantification of cell surface MHC-I in GFP+ cells from FASN^WT^ and FASN^KO^ E0771 orthotopic primary tumors. (**n**), Quantification of cell surface MHC-I in GFP+ cells from FASN^WT^ E0771 orthotopic primary tumors treated with vehicle or TVB-3166. **o,** In vitro cytotoxicity assay of luciferase-expressing FASN^WT^ or FASN^KO^ E0771 cells expressing OVA co-cultured with OT-I CD8^+^ T cells for 4 h at varying effector-to-target (E:T) ratios. Percent specific lysis, measured by BLI, is plotted across E:T ratios (n = 3 biological replicates per group). **p-r,** The effect of DOX-inducible conditional knock-down of B2M in FASN^WT^, FASN^KO^, or FASN^KO^ B2M^KD^ E0771 orthotopic models. Mice were fed DOX chow upon primary tumor palpation. (**p**), Quantification of BLI signals for lung metastases. (**q**), Flow cytometry analysis of tumor-infiltrating immune populations isolated from primary tumors, IFNγ^+^ CD8^+^ T cells. **r.** Flow cytometry analysis of circulating GFP+ tumor cells isolated from blood in FASN^WT^, FASN^KO^, or FASN^KO^ B2M^KD^ E0771 orthotopic models. Data in panels a-h,k,m-r are presented as mean ± s.e.m. Statistical significance was determined using two-tailed unpaired Student’s t-tests, a one-way ANOVA or a two-way ANOVA for comparisons between groups. Exact P values are indicated in the figures. A P value <0.05 was considered statistically significant.

Flow cytometric profiling of CD45^+^ immune cells from orthotopic tumors showed no significant differences in overall CD8^+^ or CD4^+^ T cell abundance between within FASN^WT^ and FASN^KO^ tumors (Extended Data Fig. 6a-b). However, FASN-deficiency enhanced CD8+ T cell effector phenotypes, as evidenced by increased frequencies of CD44^+^CD62- effector-like CD8+ T cells that expressed IFNγ, TNFα, granzyme B, along with increased PD-1 expression (Fig. 3a-d). Concurrently, we observed a significant reduction in polymorphonuclear myeloid-derived suppressor cells (PMN-MDSCs) (Fig. 3e). Other populations, including CD4^+^ regulatory T cells, total macrophages, dendritic cells (cDC1 and cDC2), and most NK cells, remained largely unchanged (Extended Data Fig. 6c-j). Tumor-resident NK cells showed a reduction in the terminally differentiated CD27⁻CD11b^+^ subset (Extended Data Fig.6k-l), consistent with a shift towards a more immunostimulatory tumor microenvironment. Pharmacologic FASN inhibition with TVB3166 induced similar immune changes, including more activated effector CD8^+^ T cells, and higher PD-1 expression (Fig. 3f-h).

**Extended Data Fig. 7:**
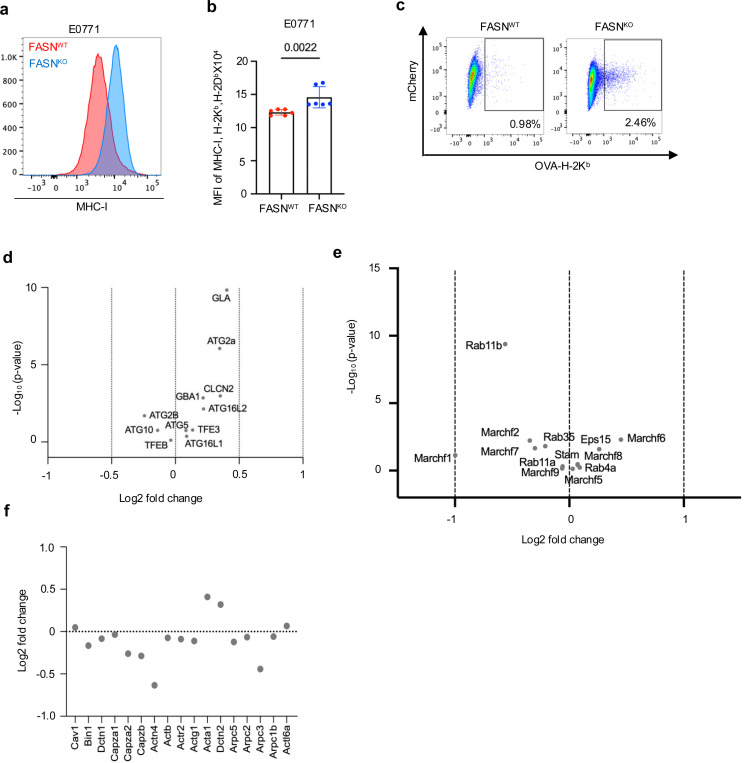
FASN loss post-translationally enhances MHC-I antigen presentation and immune recognition. **a–b,** Flow cytometry analysis of cell surface MHC-I in E0771 TNBC cells in vitro (n = 6 biological replicates per group). (**a**), Representative histograms of MHC-I expression. (**b**), quantification. **c,** Flow cytometry analysis of cell surface OVA–MHC-I complexes in FASN^WT^ and FASN^KO^ E0771 cells expressing OVA. **d–e,** Transcriptomic analysis of differentially expressed genes (DEGs) associated with MHC-I in FASN^WT^ versus FASN^KO^ tumors from orthotopic E0771 models (n = 5 biological replicates per group), including (**d**), lysosomal degradation pathways (**e**) endocytic recycling and intracellular membrane trafficking pathway. **f,** Proteomic analysis of protein expressions associated with F-actin–related pathways. Data in panels b, d-e,f are presented as mean ± s.e.m. Statistical significance was determined using two-tailed unpaired Student’s t-tests for comparisons between two groups. Exact P values are indicated in the figures. A P value <0.05 was considered statistically significant.

These immune changes suggested that lipogenic signaling suppresses tumor cell antigen presentation during metastatic progression. Transcriptomic analysis revealed no significant changes in MHC-I antigen presentation gene signatures^12,13^ upon FASN loss (Fig. 3i). In contrast, proteomic analysis revealed increased abundance of MHC-I components and the antigen processing machinery in FASN-deficient tumors (Fig. 3j), pointing to post-translational regulation. We confirmed this with qPCR, which showed that mRNA levels of H2-K1, H2-D1, and B2M remained unchanged (Fig. 3k). Indeed, both genetic and pharmacologic inhibition of FASN significantly increased cell surface MHC-I levels in both human and murine TNBC cells in vitro (Fig. 3l and Extended Data Fig. 7a-b). Importantly, OCT4/SOX2-GFP^+^ metastatic cells isolated from orthotopic tumors in immunocompetent mice also showed higher surface MHC-I expression following FASN inhibition (Fig. 3m-n).

Together these findings identify lipogenic signaling as a tumor intrinsic mechanism that suppresses cell surface MHC-I expression through post-translational mechanisms. Inhibition of FASN relieves this suppression, enhances antigen presentation on metastatic cells, and promotes a more immunostimulatory tumor microenvironment.

### Restoration of MHC-I expression following suppression of lipogenic signaling enhances CD8^+^ T cell-mediated anti-tumor immunity.

Since inhibition of lipogenic signaling restores MHC-I surface expression, we next tested whether this change improves tumor cell recognition by cytotoxic T cells. FASN-deficient E0771 cells showed increased presentation of the OVA peptide SIINFEKL)/H-2Kᵇ complex (Extended Data Fig. 7c) and triggered significantly stronger CD8^+^ T cell activation in co-culture assays (Fig. 3o). These results demonstrate that lipogenesis-dependent suppression of MHC-I directly limits tumor antigen presentation and restricts CD8^+^ T cell activation.

**Extended Data Fig. 8 |:**
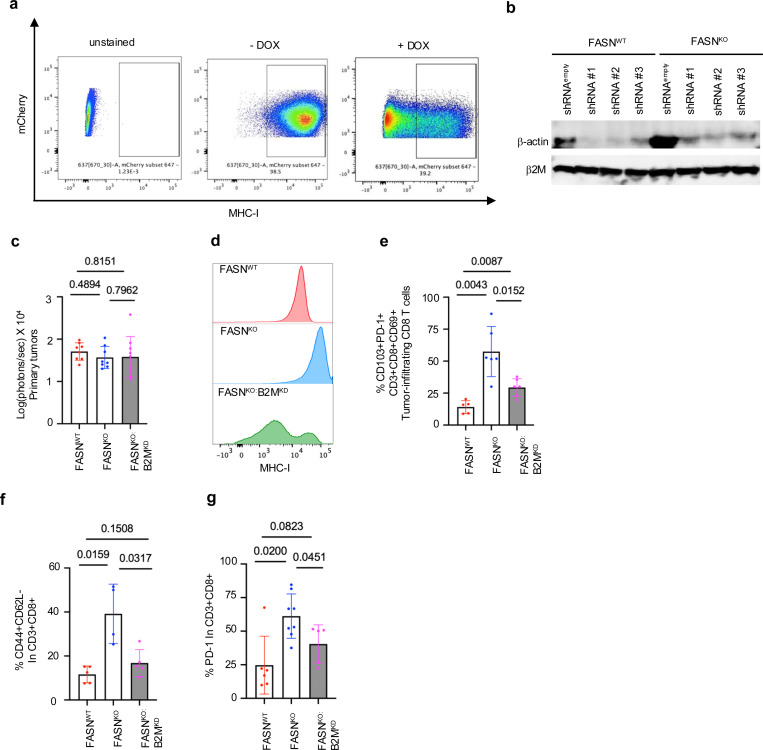
B2M knockdown reverses FASN loss–mediated immune activation and restores metastasis in orthotopic mouse models. **a,b,** Generation of FASN^WT^ and FASN^KO^ E0771 cells expressing doxycycline (DOX)–inducible B2M knockdown (B2M^KD^) in vitro. (**a**), Flow cytometry analysis of cell surface MHC-I expression in E0771 cells treated with DOX versus DMSO. (**b**) Western blot validation of B2M knockdown. **c-g,** The effect of DOX-inducible conditional B2M knock-down in FASN^WT^, FASN^KO^, and FASN^KO^ B2M^KD^ E0771 orthotopic models. Mice were fed DOX chow upon primary tumor palpation (n = 8 biological replicates per group). (**c**), Quantification of BLI signals, primary tumors. (**d**), Flow cytometry analysis showing histogram of cell surface MHC-I expression across indicated groups. (**e-g**), Flow cytometry analysis of tumor-infiltrating immune populations from primary tumors, including (**e**), CD8^+^ T cell infiltration, (**f**), central memory CD8^+^ T cells, (**g**) PD-1^+^ CD8^+^ T cells. Data in panels c-e, f-g are presented as mean ± s.e.m. Statistical significance was determined using a one-way ANOVA for comparisons between two groups. Exact P values are indicated in the figures. A P value <0.05 was considered statistically significant.

To establish causality in vivo, we generated a doxycycline-inducible conditional *B2M* knockdown system (Extended Data Fig. 8a-b). Knocking down B2M to reduce MHC-I levels largely rescued metastatic outgrowth and reversed CD8^+^ T cell activation caused by FASN deficiency (Fig. 3p-q and Extended Data Fig. 8c-g). In addition, FASN loss markedly reduced the number of circulating tumor cells (CTCs) derived from metastatic-competent populations, and this reduction was rescued by MHC-I depletion (Fig. 3r).

Together, these data establish lipogenic signaling as a tumor-intrinsic immunometabolic checkpoint. It suppresses MHC-I antigen presentation through post-translational mechanisms, restrains CD8^+^ T cell-mediated immune surveillance, and promotes metastatic dissemination in TNBC.

### EGFR palmitoylation sustains a lipid-dependent membrane signaling state to PI3K-AKT-mTOR activation and MHC-I suppression.

Previous studies have shown that MHC class I surface stability is often regulated by lysosomal degradation, ubiquitination, endosomal recycling, and altered membrane trafficking^13,14^. However, RNA-seq analysis of sorted metastatic cells from FASN-deficient orthotopic tumors revealed no significant changes in gene signatures related to these pathways (Fig. 3i and Extended Data Fig. 7d-e). F-actin levels which can influence MHC-I membrane stability^15^, were also unchanged (Extended Data Fig. 7f). These findings suggested that lipogenic signaling regulates MHC-I through a distinct post-translational mechanism.

We previously observed that metastatic cells with high lipogenic activity display elevated PI3K-AKT-mTOR signaling/mTORC1 signaling (Extended Date Fig. 1a and Fig. 1b). Given that EGFR is a key upstream regulator of this pathway, and can undergo S-palmitoylation, we hypothesized that FASN-derived palmitate promotes EGFR palmitoylation to sustain pro-metastatic signaling in TNBC. Prior work in other cancers has shown that palmitate can drive EGFR palmitoylation^16^, which influences membrane organization and signaling durability^17^.

**Extended Data Fig. 9 |:**
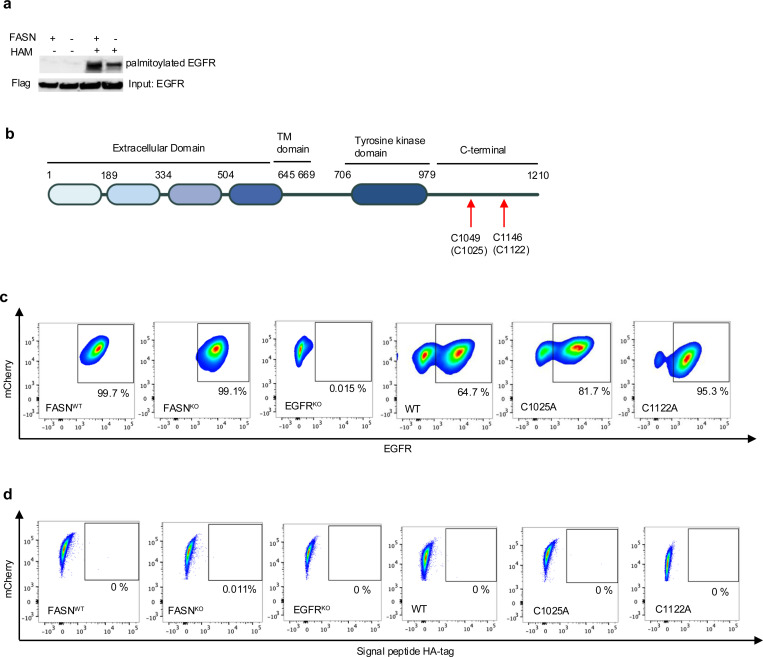
Palmitoylation-resistant EGFR mutants do not impair EGFR transmembrane formation. **a,** Western blot analysis for acyl-biotin exchange (ABE) assay of in vitro biochemical analyses using transduced EGFR^WT^ in FASN^WT^ and FASN^KO^ cells to assess EGFR palmitoylation. **b,** Schematic of EGFR domain structure. The first 23 amino acids at the N-terminus form a signal peptide. Once EGFR precursor protein is properly localized, this sequence is cleaved, releasing the peptide. Palmitoylation sites are located at cysteine residues C1049 and C1146 in the full-length protein, corresponding to C1025 and C1122 in the signal peptide–cleaved (mature) EGFR. **c,d,** Flow cytometry analysis of cell surface EGFR in MDA-MB-231 cells in vitro. (**c**) Representative plots of EGFR expression and (**d**) HA-tagged EGFR expression. FASN^WT^, FASN^KO^, and EGFR^KO^ MDA-MB-231 cells were used as controls.

To test this, we performed an acyl-biotin exchange (ABE) assay^18^ to measure S-palmitoylation of EGFR. Palmitoylated EGFR was abundant in FASN^WT^ cells but markedly reduced in FASN^KO^ cells (Extended Data Fig. 9a). This decrease in EGFR palmitoylation was accompanied by reduced downstream PI3K-AKT-mTOR signaling, whereas MAPK signaling remained unchanged (Fig. 2f). Prior work has shown that palmitoylated EGFR preferentially interacts with the PI3K regulatory subunit PIK3R1 (p85), enhancing PI3K recruitment and activation^19^. These results suggest that MAPK does not compensate for the loss of palmitoylated EGFR in TNBC. Together, these findings support a model in which lipogenesis-dependent EGFR palmitoylation organizes a membrane-associated signaling scaffold that stabilizes PI3K-mTOR activation during metastatic progression.

Palmitoylation of EGFR occurs at specific C-terminal cysteine residues (C1025, C1034, C1122) mediated by the palmitoyltransferases ZDHHC20 and ZDHHC13^19,20^. To directly assess the functional importance these sites, we generated EGFR knockout cells and reconstituted them with either wild-type EGFR or palmitoylation-resistant mutants. The mutant receptors localized normally to the cell surface (Extended Data Fig. 9c-d), indicating that palmitoylation is not required for EGFR trafficking or membrane insertion.

**Extended Data Fig. 10 |:**
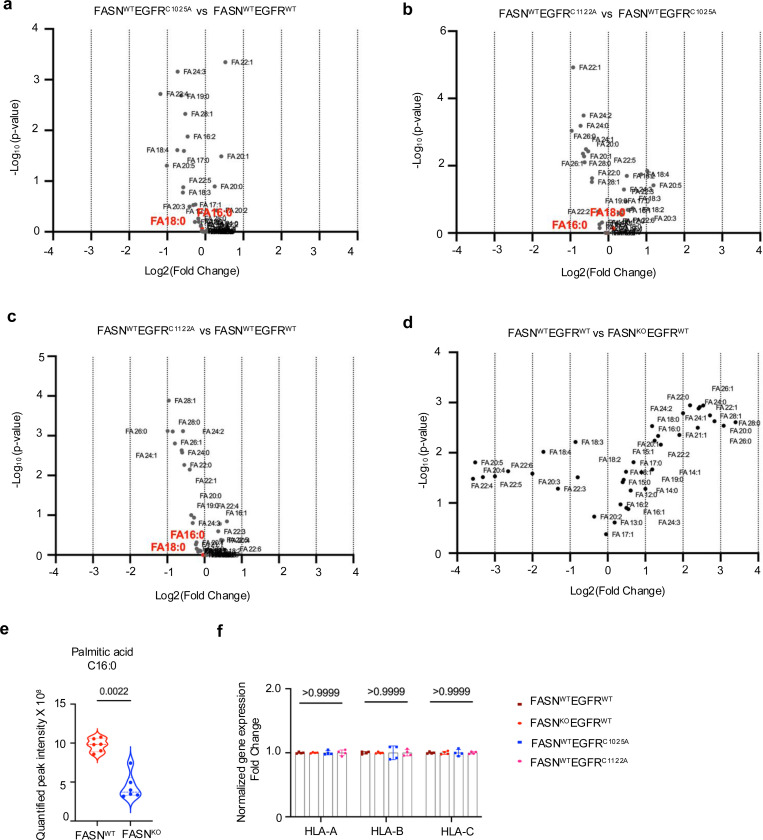
EGFR palmitoylation functions downstream of de novo lipid synthesis and does not alter MHC-I mRNA expression. **a–d,** LC–MS–based lipidomic profiling of fatty acid abundances in EGFR^WT^ versus palmitoylation-resistant EGFR mutants and FASN^WT^ versus FASN^KO^ MDA-MB-231 cells in vitro (n = 4 biological replicates per group). Cells were cultured in complete media. (**a**) FASN^WT^EGFR^C1025A^ versus FASNWTEGFRWT (**b**) FASNWTEGFRC1122A versus FASNWTEGFRC1025A (**c**) FASNWTEGFRC1122A versus FASN^WT^EGFR^WT^ (**d**) FASN^WT^EGFR^WT^ versus FASN^KO^EGFR^WT^. **e,** LC–MS analysis of palmitic acid levels in FASN^WT^ and FASN^KO^ E0771 cells in vitro (n = 4 biological replicates per group). **f.** Quantification of mRNA expression of MHC-I subunits in MDA-MB-231 cells expressing FASN^WT^ EGFR^WT^, FASN^KO^ EGFR^WT^, FASN^WT^ EGFR^C1025A^, and EGFR^WT^ EGFR^C1122A^ in vitro. Data in panels a-d,e,f are presented as mean ± s.e.m. Statistical significance was determined using two-tailed unpaired Student’s t-tests or a two-way ANOVA for comparisons between two groups. Exact P values are indicated in the figures. A P value <0.05 was considered statistically significant.

**Fig. 4 |:**
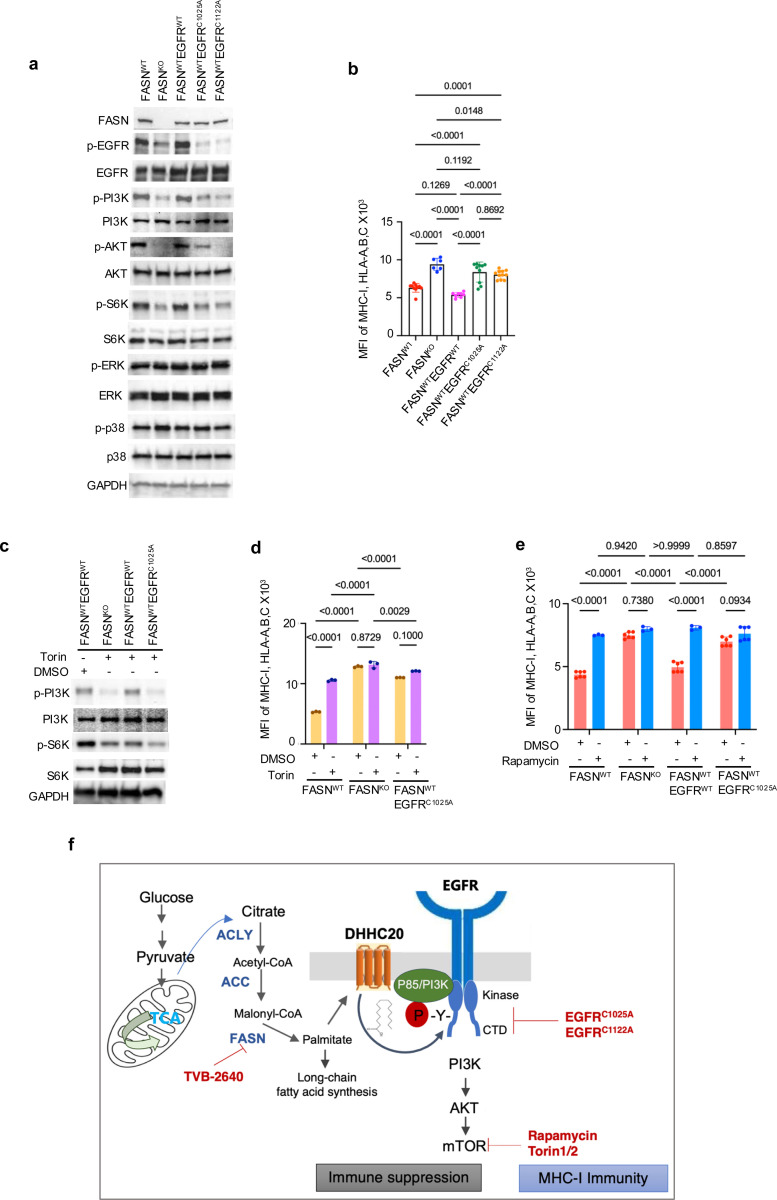
EGFR palmitoylation drives a lipid-dependent membrane signaling state through PI3K-AKT-mTOR signaling to suppress MHC-I-immune recognition. **a,** Western blot analysis of EGFR signaling in MDA-MB-231 cells expressing FASN^WT^, FASN^KO^, FASN^WT^EGFR^WT^,FASN^WT^EGFR^C1025A^, and EGFR^WT^ EGFR^C1122A^ **b,** Quantification of flow cytometry analysis of cell surface MHC-I with the same indicated genotypes (n = 5 biological replicates per group). **c,d,** Effects of Torin on MHC-I expression and EGFR signaling in MDA-MB-231 cells expressing FASN^WT^, FASN^KO^, and FASN^WT^EGFR^C1025A^ (n = 3 biological replicates per group). (**c**) Flow cytometry quantification of cell surface MHC-I in MDA-MB-231 cells (FASN^WT^, FASN^KO^, and FASN^WT^EGFR^C1025A^) treated with DMSO or Torin (100 nM) for 48 hours. (**d**) Western blot analysis of EGFR signaling in the same indicated genotypes. **e,** Quantification of flow cytometry analysis of cell surface MHC-I in MDA-MB-231 cells (FASN^WT^, FASN^KO^, FASN^WT^EGFR^WT^,and FASN^WT^EGFR^C1025A^) treated with DMSO or rapamycin (20 nM) for 48 hours (n = 5 biological replicates per group). **f.** Schematic of lipogenesis and immune modulation downstream of EGFR signaling. Glucose metabolism generates citrate, which is converted to acetyl-CoA by ACLY and subsequently to malonyl-CoA by ACC. Palmitate, generated by FASN, undergoes desaturation and elongation. Through DHHC20 palmitoyltransferase, palmitate promotes EGFR palmitoylation, facilitating recruitment of the PI3K subunit p85 and activation of PI3K/AKT/mTOR signaling, leading to regulation of MHC-I expression and tumor immunity. Key inhibitors are shown in red, and major pathways are indicated by arrows. Data in panels b,c,d are presented as mean ± s.e.m. Statistical significance was determined using a one-way ANOVA or a two-way ANOVA for comparisons between groups. Exact P values are indicated in the figures. A P value <0.05 was considered statistically significant.

Importantly, expression of palmitoylation-deficient EGFR suppressed PI3K-AKT-mTOR signaling without altering MAPK signaling (Fig. 4a) and fully phenocopied the effects of FASN deficiency by restoring cell surface MHC-I expression (Fig. 4b), while MHC-I mRNA levels remained unchanged (Extended Data Fig. 10f). These mutants did not alter free fatty acid levels (Extended Data Fig. 10a-e), confirming that EGFR palmitoylation functions downstream of de novo lipid synthesis.

Collectively, these findings identify EGFR palmitoylation as a lipid-dependent membrane signaling scaffold that couples de novo fatty acid synthesis to sustained PI3K-AKT-mTOR activity and post-translational suppression of MHC-I antigen presentation, and metastatic immune escape.

### mTOR is required for post-translational suppression of MHC-I.

The PI3K-AKT-mTOR pathway has been implicated in MHC-I regulation through multiple mechanisms including AKT-driven ubiquitination and lysosomal degradation, as well as mTORC1-dependent control of receptor trafficking and surface stability^21,22^. However, RNA-seq analysis of FASN-deficient metastatic cells showed no significant changes in lysosomal, ubiquitination, or MHC-I trafficking. These data suggested that lipogenic signaling suppresses MHC-I through a distinct mechanism downstream of PI3K-AKT signaling and prompted us to test the role of mTOR.

**Extended Data Fig. 11 |:**
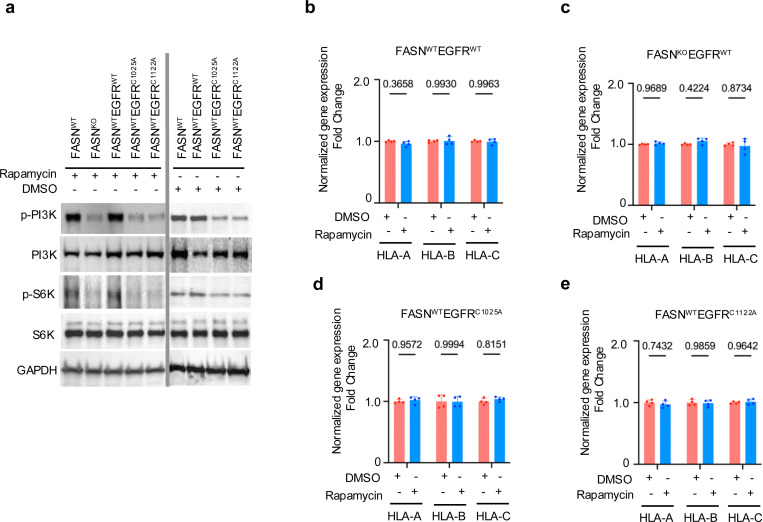
Pharmacologic inhibition of mTORC1 recapitulates MHC-I expression induced by FASN deficiency or palmitoylation-resistant EGFR mutants. **a,** Western blot analysis of EGFR signaling in MDA-MB-231 cells (FASN^WT^, FASN^KO^, FASN^WT^EGFR^WT^, FASN^WT^EGFR^C1025A^, and EGFR^WT^ EGFR^C1122A^) treated with DMSO or rapamycin (20 nM) for 48 hours. **b–e,** Quantification of MHC-I subunit mRNA expression in MDA-MB-231 cells expressing (**b**), FASN^WT^EGFR^WT^, (**c**), FASN^KO^EGFR^WT^, (**d**), FASN^WT^ EGFR^C1025A^, and (**e**) EGFR^WT^ EGFR^C1122A^. (n = 4 biological replicates per group).

Pharmacologic inhibition of Torin1, a catalytic mTOR inhibitor that blocks both mTORC1 and mTORC2, reduced downstream S6K phosphorylation (Fig. 4c) and significantly increased cell surface MHC-I expression (Fig. 4d). Similar restoration of MHC-I surface expression was observed with rapamycin, reinforcing the central requirement for mTOR activity (Fig. 4e and Extended Data Fig. 11a), without altering MHC-I mRNA transcripts (Extended Data Fig. 11b-e). Together, these findings identify mTOR signaling as a critical downstream effector of the lipogenesis-EGFR axis that suppresses MHC-I surface expression through a post-transcriptional mechanism.

**Extended Data Fig. 12 |:**
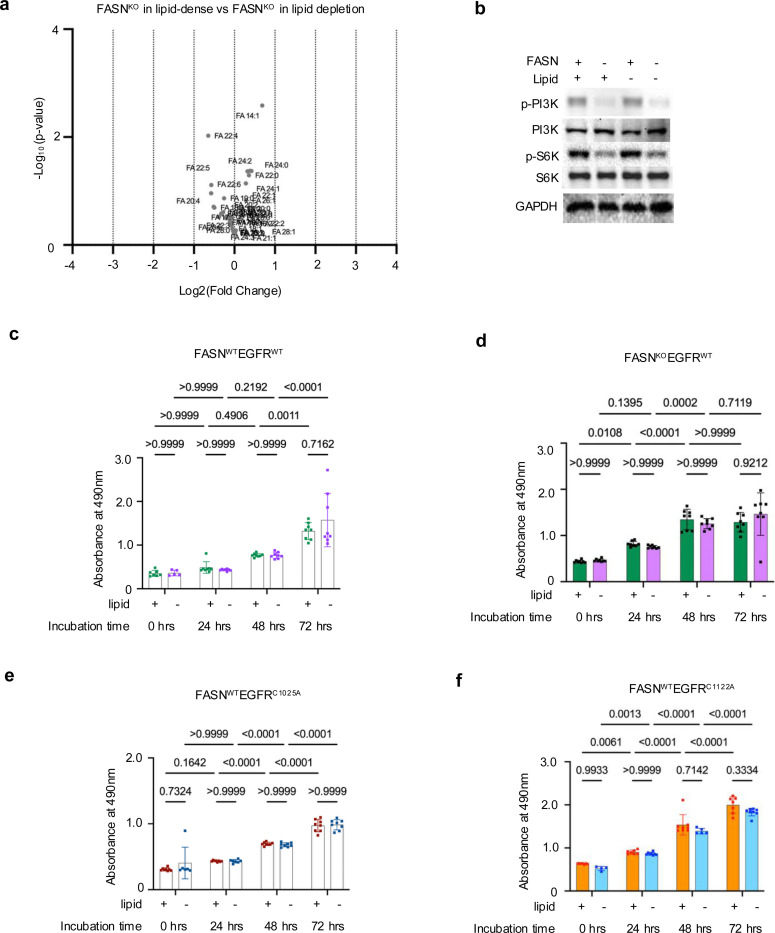
EGFR signaling through palmitoylation relies on FASN-mediated fatty acid synthesis. **a,** Volcano plot of LC–MS–based lipidomic analysis comparing fatty acid abundance in FASN^KO^ MFA-MB-231 cells cultured in lipid-rich (lipid-dense FBS) versus lipid-depleted FBS conditions in vitro (n = 4 biological replicates per group). **b,** Western blot analysis of EGFR downstream signaling in FASN^WT^ and FASN^KO^ MDA-MB-231 cells treated with lipid-dense or lipid-depleted FBS. **c–f,** In vitro cell proliferation assays of MDA-MB-231 cells cultured under lipid-dense or lipid-depleted FBS conditions at 0, 24, 48, and 72 h, including (**c**) FASN^WT^EGFR^WT^ (**d**) FASN^KO^ EGFR^WT^ (**e**) FASN^WT^ EGFR^C1025A^ (**f**) EGFR^WT^ EGFR^C1122A^. (n = 8 biological replicates per group). Data in panels a, c-f are presented as mean ± s.e.m. Statistical significance was determined using two-tailed unpaired Student’s t-tests or a two-way ANOVA for comparisons between two groups. Exact P values are indicated in the figures. A P value <0.05 was considered statistically significant.

To test whether exogenous lipids could bypass the need for de novo lipogenesis, we cultured FASN-deficient cells in lipid-rich conditions. Notably, FASN^KO^ cells failed to restore intracellular palmitate or total free fatty acid levels even in the presence of abundant exogenous lipids (Extended Data Fig. 12a and Extended Data Fig. 10d-e). Consistent with this, EGFR downstream signaling remained suppressed and cell proliferation was unaffected by lipid supplementation (Extended Data Fig. 12b-f). These findings demonstrate that metastatic TNBC cells depend predominantly on endogenous (de novo) lipid synthesis rather than exogenous lipid uptake to sustain EGFR signaling and metastatic fitness.

## Discussion

Despite advances in immune checkpoint blockade, TNBC remains largely refractory to immunotherapy, in part due to tumor-intrinsic suppression of MHC-I antigen presentation^23^. Here, we identify a previously unrecognized lipogenesis-dependent immunometabolic program that drives metastatic immune escape in TNBC. Specifically, FASN-mediated de novo palmitate synthesis promotes EGFR palmitoylation, thereby establishing a membrane-associated scaffold that sustains PI3K-AKT-mTOR signaling and post-translationally suppresses MHC-I surface expression (Fig. 4f).

Notably, this lipid-dependent EGFR-PI3K-mTOR axis operates independently of MAPK signaling. In ovarian cancer, FASN inhibition suppresses PI3K-AKT-mTORC1 but triggers compensatory MAPK activation^24^. In striking contrast, MAPK signaling failed to compensate for the loss of palmitoylated EGFR in TNBC. This difference likely arises because palmitoylated EGFR preferentially recruits the PI3K regulatory subunit PIK3R1 (p85) over Grb2, thereby favoring PI3K signaling while limiting MAPK pathway engagement. This biased signaling is further amplified in the context of oncogenic KRAS, which is common in aggressive TNBC subsets. Consequently, disruption of EGFR palmitoylation selectively impairs the PI3K-mTOR arm without compensatory reactivation of MAPK, revealing a non-redundant, context-specific vulnerability in metastatic TNBC.

Our findings also refine current models of lipid metabolism in metastasis. While prior studies have emphasized adaptive lipid scavenging and organ-specific lipid availability^25^, we demonstrate that metastatic TNBC cells maintain a selective dependence on de novo palmitate synthesis to sustain EGFR palmitoylation and immune-suppressive signaling, even in lipid-rich lung microenvironment. Exogenous lipids failed to rescue palmitate levels, EGFR signaling, or metastatic fitness in FASN-deficient cells.

By showing that tumor-derived palmitate drives EGFR palmitoylation, our work establishes palmitoylation as a critical regulator of membrane signaling organization during metastatic immune escape. This creates a lipid-dependent signaling scaffold that maintains PI3K-mTOR activity and suppresses MHC-I through post-translational mechanisms. This pathway operates independently of canonical MHC-I trafficking or lysosomal degradation and is distinct from EGFR-MAPK-mediated transcriptional repression of MHC-I via IRF1 and STAT1^26^.

Our data also clarify a context-specific role for mTOR in immune regulation. Whereas mTORC1 inhibition enhances MHC-I expression in some settings^27,28^, we show that in metastatic TNBC cells, mTOR activity downstream of palmitoylated mediates EGFR promotes post-translational suppression of MHC-I surface stability.

Therapeutically, these findings have important implications. Pharmacologic FASN inhibition using agents which are already in clinical trials, may selectively impair metastatic progression by restoring anti-tumor CD8^+^ T cell immunity.

In summary, this study identifies lipogenesis-dependent EGFR palmitoylation as a tumor cell-intrinsic immunometabolic checkpoint that couples fatty acid synthesis to metastatic competence and immune evasion. By revealing a druggable link between lipid metabolism, membrane signaling, and antigen presentation, our work identifies a promising therapeutic opportunity to disable metastasis and reactivate anti-tumor immunity in TNBC.

## Methods.

### Cell lines and culture conditions

MDA-MB-231-LM2 and EO771 cells were cultured in DMEM supplemented with 10% FBS, 1% L-glutamine, 1% PS, and 2 mM HEPES as described^7,8,10^. EO771-ML1 line with increased lung metastasis potential was generated^8^. MDA-MB-453 cells (ATCC, Manassas, VA, USA) were cultured in DMEM with 10% FBS, 1% L-glutamine, and 1% penicillin-streptomycin (PS). HCC1806 cells (gift from Dr. Sophia Ran, Southern Illinois University) were maintained in RPMI with 10% FBS, 1% L-glutamine, and 1% PS. NSCLC cell lines H358 (gift from Dr. Ding Cheng Gao, Weill Cornell Medicine), mEGFRdel19.2 (gift from Dr. Lynn Heasley, University of Colorado Anschutz Medical Campus), and HKP1^29^ were cultured in RPMI with 10% FBS, 1% L-glutamine, and 1% PS. Reagents are listed in Supplementary Table 4.

### CRISPR/Cas9-mediated gene knockout.

FASN knockout in E0771 cells was generated using CRISPR/Cas9 ribonucleoprotein (RNP) electroporation (Neon Transfection System, Thermo Fisher Scientific)^30^. sgRNA duplexes were formed by annealing ATTO-647–tracrRNA with target-specific crRNAs (IDT), followed by assembly with Cas9 protein at a 1:1.2 molar ratio. Cells (1 × 10⁶) were resuspended in Neon Buffer R, mixed with RNP complexes and electroporation enhancer, and electroporated (1,400 V, 10 ms, 4 pulses). Cells were recovered in complete medium, and knockout efficiency was assessed 18 h post-transfection. Single cells were sorted by flow cytometry, clonally expanded, and validated by Western blot. For stable FASN or EGFR knockout in MDA-MB-231 cells, gene-targeting gRNAs were cloned into the Lenti-Cas9-gRNA-TagBFP2 vector (Addgene #124774)^31^. Lentiviral particles were produced in HEK293 cells and used to transduce target cells. Knockout clones were isolated by flow cytometry and validated by Western blot. CRISPR guides, reagents, and antibodies are listed in Supplementary Table 3, 4 and 6.

### Lentiviral generation and transduction of EGFR constructs.

Palmitoylation-deficient EGFR mutants were generated from wild-type EGFR (Addgene #179263) using the Q5 Site-Directed Mutagenesis Kit. Plasmids were purified using the NucleoBond Xtra Midi Plus EF kit. Lentiviral particles were produced in HEK293T cells by calcium phosphate transfection, and viral supernatants were collected 48–72 h post-transfection and filtered (0.45 μm). EGFR-knockout MDA-MB-231 cells were transduced with viral supernatant in the presence of polybrene (10 μg/mL) and selected with puromycin (0.5 μg/mL). Cells were maintained for 1-week post-selection, and EGFR expression was validated followed by enrichment via flow cytometry. DNA cloning and molecular biology reagents are listed in Supplementary Table 7.

### Lentiviral generation of conditional B2M knock-down.

For doxycycline-inducible MHC-I knockdown, B2M shRNA was cloned into the pLV[miR30]-TRE3G>EBFP-miR30-shRNA vector (VectorBuilder) using XbaI/SpeI. FASN^WT^ or FASN^KO^ E0771 cells were transduced, selected with 10 μg/mL blasticidin in 1 μg/mL doxycycline, and enriched by FACS based on EBFP expression and reduced surface MHC-I. Knockdown was confirmed by Western blot. DNA cloning and molecular biology reagents are listed in Supplementary Table 7.

### In vitro cell proliferation and signaling assays.

Cell proliferation was measured using the CellTiter 96^®^ AQueous One Solution Cell Proliferation Assay (MTS; Promega, G3582) according to the manufacturer’s instructions. Cells were seeded at 5,000 cells per well in 96-well plates in complete medium and incubated under the indicated conditions. Absorbance at 490 nm was measured at the specified time points using a SpectraMax^®^ M5 multi-mode microplate reader (Molecular Devices). All experiments were performed with at least three independent biological replicates. For assessment of FASN-dependent proliferation, FASN^WT^ and FASN^KO^ cells were seeded under identical conditions and incubated for 72 h in complete medium prior to MTS-based quantification. For lipid-dependent proliferation assays, cells were cultured in complete medium supplemented with either lipid-dense or lipid-depleted fetal bovine serum (FBS) for up to 72 h. Cell proliferation was quantified using the MTS assay as described above. Absorbance at 490 nm was recorded using a SpectraMax^®^ M5 microplate reader. For pharmacologic inhibition studies, cells were seeded at 5,000 cells per well in 96-well plates and treated the following day with TVB3166 or TVB2640 (3 days), or rapamycin or Torin (2 days), with DMSO control. Cells were incubated for the indicated durations prior to MTS-based measurement of cell proliferation. For parallel signaling analyses, cells were seeded in 6-well plates under identical conditions as proliferation assays and harvested for Western blot analysis of EGFR downstream signaling pathways. Reagents are listed in Supplementary Table 4.

### In vitro invasion assay following FASN knockout.

Cells were seeded in serum-free DMEM into Matrigel-coated invasion inserts (BD BioCoat), with 10% FBS-containing DMEM in the lower chamber as a chemoattractant. After 24 h at 37 °C, non-invading cells were removed from the upper surface, and invaded cells on the underside were fixed and stained using Kwik-Diff Stains (Thermo Fisher Scientific). Invaded cells were imaged in six random fields per insert and quantified by phase-contrast microscopy, with values normalized to control conditions. Experiments were performed in at least three independent biological replicates. Reagents are listed in Supplementary Table 4.

### In vitro cell cycle analysis following FASN knockout.

Cell cycle distribution was assessed using an Abcam flow cytometry kit. Cells were fixed in ice-cold 70% ethanol, washed, and stained with propidium iodide (PI) and RNase A for 30 min at room temperature in the dark. DNA content was analyzed by flow cytometry, and G0/G1, S, and G2/M phases were quantified based on PI fluorescence intensity. Reagents are listed in Supplementary Table 4.

### 3D bioprinting spheroid formation assay

Spheroid Formation and Analysis E0771 variants (GFP+/−, FASN WT/KO) were cultured and seeded in AggreWell^™^ 800 plates (7.50 × 10^5^ cells/well). Brightfield images were analyzed using CellProfiler (v4.2.7). Briefly, images were converted to grayscale, noise-reduced with a Gaussian filter (sigma: 2), and segmented via Sobel edge detection to define spheroid boundaries. To quantify growth while accounting for variations in spheroid compaction and density, a Normalized Opacity-Adjusted Spheroid Area were calculated. This metric, derived from the product of the spheroid area and the inverted mean intensity (opacity) and normalized to the respective control group, provides a more robust measure of 3D growth than area alone.

### Animal work

All animal procedures were approved by the Institutional Animal Care and Use Committee at Weill Cornell Medical College (protocol no. 0806–763A). Female C57BL/6J mice (The Jackson Laboratory) were used unless otherwise specified. OT-I (C57BL/6-Tg (TcraTcrb)1100Mjb/J) and Thy1.1 mice (B6.PL-Thy1a/CyJ) were obtained from The Jackson Laboratory and bred in-house. Mice were housed under standard conditions (12 h light/dark cycle, 65–75°F, 40–60% humidity) and used for in vivo experiments at 8 weeks of age.

### Orthotopic E0771 tumor model (genetic and pharmacologic studies).

Female C57BL/6J mice (n = 10/group) were orthotopically injected with 1 × 10⁵ FASN^WT^ or FASN^KO^ E0771 cells in 50 μL HBSS into the fourth mammary fat pad. In pharmacologic studies, mice received FASNWT E0771 cells. Primary tumors were allowed to grow for up to 4 weeks or until reaching 1 cm³, followed by surgical resection. Mice were then monitored weekly for lung metastasis by bioluminescence imaging (BLI) and caliper measurements for up to 3 weeks. At 7 weeks post-implantation, mice were euthanized for lung collection and downstream analyses. Survival cohorts were monitored until humane endpoints.

### In vivo MHC class I–depleted orthotopic model

Mice (n = 10/group) were injected with 1 × 10⁵ E0771 cells expressing FASN^WT^, FASN^KO^, or FASN^KO^/B2M^KD^ into the mammary fat pad. Doxycycline was administered via chow starting day 5 post-implantation. Tumors were allowed to grow up to 1 cm³ and then resected. Mice were followed longitudinally by BLI for lung metastasis and survival.

### In vivo FASN inhibitor study.

Mice bearing orthotopic FASN^WT^ E0771 tumors received daily intraperitoneal injections of TVB3166 (5 mg/kg) starting day 5 post-implantation. Tumor growth and metastasis were monitored by BLI and caliper measurements. Toxicity was assessed by body weight, clinical status, and H&E histology, with no significant adverse effects observed. Compounds are listed in Supplementary Table 4.

### Histology

Lungs were fixed in 4% paraformaldehyde for at least 48 h, processed through ethanol and xylene, and embedded in paraffin using a Leica ASP6025 tissue processor (Leica Biosystems). Sections (5 μm) were stained with hematoxylin and eosin (H&E) and evaluated by a board-certified veterinary pathologist. Tumor burden was quantified by assessing neoplastic foci in large masses, perivascular and peribronchiolar aggregates, and subpleural lesions. Slides were scanned using an Olympus VS200 slide scanner (Evident Scientific) and analyzed with OlyVIA software v3.4.1.

### CD8^+^ T cell killing assay (E0771-OVA model).

In vitro BLI-based cytotoxicity assays were established as described^33^. E0771 cells were transduced with pLVX-puro-cOVA (Addgene #135073) to generate E0771-OVA cells expressing the SIINFEKL (OVA257–264) peptide. SIINFEKL–MHC-I–positive cells were enriched by FACS using A647-conjugated antibody staining. Cells were cultured in DMEM supplemented with 10% FBS, 2 mM L-glutamine, 2 mM HEPES, and penicillin/streptomycin. CD8^+^ T cells were isolated from mouse spleens by MACS after ACK lysis and activated on plates coated with anti-CD3ε/CD28 in the presence of IL-2 (50 U/mL) for 2 days. For cytotoxicity assays, luciferase-expressing E0771-OVA cells were co-cultured with activated CD8^+^ T cells at indicated E:T ratios (1:1–20:1) for 4 h in the presence of IL-2. Tumor cell viability was assessed by bioluminescence imaging. Maximum and spontaneous lysis controls were obtained using 1% NP-40 and media alone, respectively. Percent specific lysis was calculated as: % specific lysis = 100 × (spontaneous RLU − test RLU) / (spontaneous RLU − maximal killing RLU).

### Flow cytometry analysis.

Cells were stained in FACS buffer and analyzed by flow cytometry; dead cells were excluded using DAPI. For in vivo analyses, tumors were dissociated using collagenase IV (1 mg/mL), hyaluronidase (50 U/mL), and DNase I (0.1 mg/mL), followed by TrypLE digestion and ACK lysis. Single-cell suspensions were stained with surface markers plus Fc block. Tumor cells were defined as CD45⁻ mCherry^+^ GFP^+^/GFP⁻, and immune cells as CD45^+^. Most cells (~90%) were sorted by CD45 for downstream assays. For immune profiling, CD45^+^ cells were stimulated with PMA/ionomycin in the presence of brefeldin A and monensin for 6 h, followed by surface, intracellular, and viability staining (Zombie Aqua). CTCs were isolated from blood after ACK lysis and defined as DAPI⁻ CD45⁻ CD31⁻ Ter119⁻ mCherry^+^ GFP^+^ cells. Flow cytometry was performed on BD FACSymphony S6; compensation used single-stained beads, and data were analyzed in FlowJo (v10.10.1). MESF beads were used for calibration. shRNA, antibodies, reagents are listed in Supplementary Table 2, 4, and 5.

### EGFR palmitoylation analysis by ABE assay.

EGFR constructs (Addgene #18788) were generated by Q5 site-directed mutagenesis (NEB) and expressed in FASNWT or FASNKO E0771 cells under G418 selection, with EGFR expression validated by Western blot. Cells were lysed in 20 mM NEM buffer and incubated at 4 °C, followed by protein isolation using chloroform–methanol precipitation. Protein pellets were resuspended and split into HAM− and HAM+ conditions, with HAM+ samples treated with 0.7 M hydroxylamine for 1 h to cleave thioester-linked palmitoylation. Following re-precipitation, free cysteines were labeled with biotin-HPDP. Biotinylated proteins were enriched using streptavidin–agarose beads, washed extensively, and eluted in Laemmli buffer. Samples were analyzed by SDS–PAGE to assess EGFR palmitoylation levels. Reagents and Antibodies are listed in Supplementary Table 4 and 5.

### Western blot

Cell lysates were prepared in RIPA buffer (Millipore) supplemented with PMSF and protease inhibitors. Protein concentrations were quantified using the Pierce^™^ BCA Protein Assay Kit (Thermo Scientific). Equal amounts of protein were resolved by SDS-PAGE (4–20% gels) and transferred to PVDF membranes. Membranes were blocked with 5% non-fat milk and incubated with primary antibodies followed by HRP-conjugated secondary antibodies (R&D Systems). Signals were detected using ECL Prime (Amersham). Reagents and antibodies are listed in Supplementary Table 4 and 6.

### RNA sequencing and transcriptomic analysis.

For mouse tissues and sorted tumor cells, total RNA was used to prepare libraries with the NEBNext Ultra II Directional RNA Library Prep Kit following poly(A) enrichment (NEBNext Poly(A) mRNA Magnetic Isolation Module; New England Biolabs). Libraries were sequenced on an Illumina NovaSeq X Plus platform (paired-end 2 × 51 bp). Raw BCL files were converted to FASTQ using bcl2fastq v2.20, followed by adapter trimming and quality filtering with Cutadapt v3.54^34^. Reads were aligned to the mouse genome (GRCm39) using STAR v2.7.9a^35^, and gene-level counts were generated with HTSeq-count v0.13.5^36^. Differential expression analysis was performed using DESeq2 v1.38.3^37^. Gene set enrichment analysis (GSEA) was conducted using clusterProfiler v4.6.2^38^ against MSigDB (Hallmark, curated, ontology), KEGG, and Reactome gene sets, with visualization using enrichplot and ggplot2 v3.4.1.

### RNA extraction and qRT–PCR.

Total RNA was isolated from cell lines, bulk tissues, and sorted tumor cells, according to the manufacturer’s instructions. Approximately 250 ng RNA was reverse transcribed using qScript cDNA SuperMix (QuantaBio). Quantitative RT-PCR was performed using SsoAdvanced SYBR Green Supermix (Bio-Rad) on a CFX96 Real-Time PCR system. All reactions were run in triplicate, and specificity was confirmed by melt-curve analysis. Gene expression was normalized to GAPDH and calculated using the 2^−ΔCt method. Primer sequences are listed in Supplementary Table 1.

### LC-MS–based lipid and metabolite profiling.

Free fatty acids, lipids, and short-chain acyl-CoAs were extracted using cold organic solvents, clarified by centrifugation and dried under vacuum^44^. Samples were reconstituted in appropriate LC-MS solvents prior to analysis. Separation was performed on a Vanquish UHPLC system (Thermo Scientific) equipped with a Cadenza CD-C18 column coupled to a Q Exactive Orbitrap mass spectrometer. For free fatty acids, a methanol/isopropanol–based gradient was used, and data were acquired in negative ion mode (m/z 140–1000, resolution 70,000). Lipidomics employed data-dependent MS/MS acquisition in both ion modes (MS1 resolution 70,000; MS2 resolution 17,500). Short-chain acyl-CoAs were analyzed in positive ion mode using a methanol-based gradient (2–95% B over 8 min). Metabolites and lipids were identified by accurate mass (≤5 ppm) and retention time matching. Lipids were additionally quantified using MS-DIAL v4.9, while other metabolites were quantified by MS signal intensity using XCalibur 4.1.

### Global proteomics analysis.

#### FASP digestion.

Mouse breast tumor tissues were lysed by ultrasonication and centrifuged at 12,000 × g for 10 min at 4 °C. Supernatants were transferred to Microcon YM-10 filter devices (Millipore) and centrifuged at 12,000 × g for 10 min. Proteins were washed twice with 50 mM ammonium bicarbonate, reduced with 10 mM dithiothreitol (DTT) at 56 °C for 1 h, and alkylated with 20 mM iodoacetamide (IAA) at room temperature in the dark for 1 h. Samples were digested overnight at 37 °C with trypsin (1:50 enzyme-to-protein ratio). Peptides were collected by centrifugation, washed once, lyophilized, and resuspended in 0.1% formic acid for LC–MS/MS analysis.

#### Nano LC–MS/MS analysis.

Peptides were analyzed on an Ultimate 3000 HPLC system (Dionex) coupled to an Easy-nLC 1000 (Thermo Fisher Scientific) with a C18 column (1.8 μm, 0.15 × 100 mm) at 600 nL/min. Mobile phase A was 0.1% formic acid in water, and B was 90% acetonitrile. The gradient was 4–7% B (2 min), 7–25% B (78 min), 25–35% B (4 min), and 35–90% B (2 min). MS data were acquired over 350–1,600 m/z at 30,000 resolution using HCD with AGC 1 × 10⁵, normalized collision energy 35%, isolation window 1.0 Th, and 30 s dynamic exclusion. Data analysis. Raw files were processed in MaxQuant (v2.1) against the *Mus musculus* database. Carbamidomethylation (C) was set as a fixed modification; oxidation (M) and N-terminal acetylation were variable modifications. Trypsin specificity allowed up to two missed cleavages. Precursor and fragment mass tolerances were 20 ppm and 0.05 Da, respectively. Differentially expressed proteins were defined as FC > 2 (upregulated) or < 0.5 (downregulated).

### Statistical analysis

Unless otherwise stated, all experiments were performed at least twice with consistent results. Statistical analyses were conducted using GraphPad Prism (v11.0.1). Comparisons between two groups were performed using a two-tailed Student’s t-test. For multiple group comparisons, one-way ANOVA with Tukey’s post hoc test was used, and for experiments with two independent variables, two-way ANOVA with Tukey’s multiple comparisons test was applied. Error bars represent mean ± SEM from independent biological replicates. Tumor burden measured by bioluminescence imaging (BLI) was analyzed using two-way ANOVA with Šídák’s multiple comparisons correction, with outliers identified using Grubbs’ test (α = 0.0001). Overall survival was assessed using the log-rank (Mantel–Cox) test. Survival studies included ≥10 mice per group, providing 95% power to detect ≥20% differences in survival at α = 0.05. Metabolomics data were analyzed using multiple unpaired two-tailed t-tests. A P value < 0.05 was considered statistically significant.

## Extended Data

**Extended Data Fig. 13 |:**
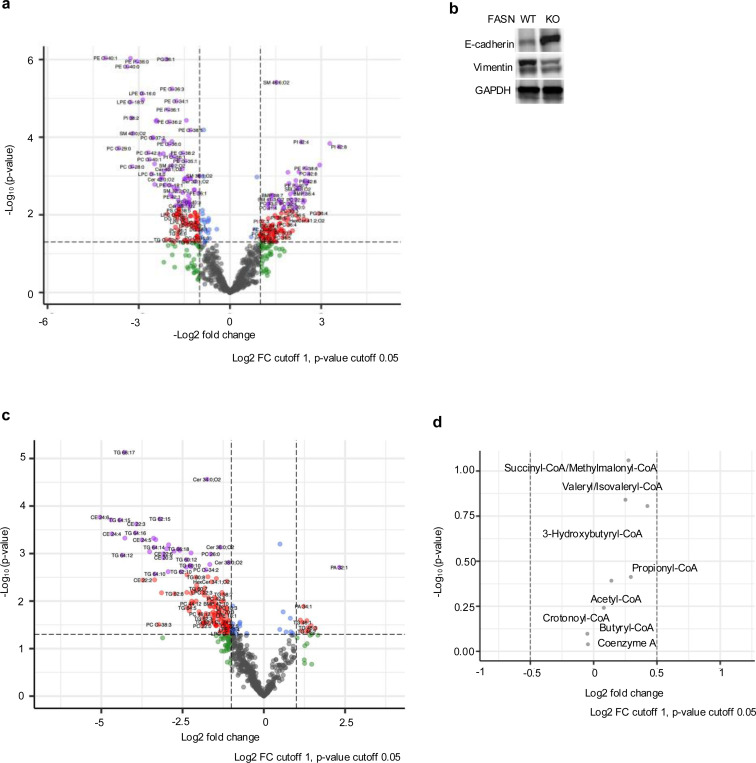
Metastasis-initiating cells rely on lipogenesis-dependent outgrowth. **a,** Volcano plot of LC–MS–based lipidomic analysis comparing lipid abundance in FASN^WT^ versus FASN^KO^ primary tumors (n = 4 biological replicates per group). **b,** Western blot analysis of FASN^WT^ and FASN^KO^ tumor cells isolated from orthotopic primary tumors. **c,d,** LC–MS–based lipidomic profiling of FASN^KO^ versus FASN^WT^ lung metastases (n = 4 biological replicates per group), including (**c**), lipid abundance and (**d**) short-chain acyl-CoA levels. Data in panels a, c-f are presented as mean ± s.e.m. Statistical significance was determined using two-tailed unpaired Student’s t-tests or a two-way ANOVA for comparisons between two groups. Exact P values are indicated in the figures. A P value <0.05 was considered statistically significant.

## Supplementary Material

Supplement 1
