# Supplementary material for: Lipogenesis-driven EGFR palmitoylation enables metastatic immune evasion in triple-negative breast cancer
Source: bioRxiv. 2026 May 26:2026.05.21.726063. Preprint. [Version 1] doi: 10.64898/2026.05.21.726063 (PMC13232100; doi:10.64898/2026.05.21.726063)
Supplement: Supplement 1 [file NIHPP2026.05.21.726063v1-supplement-1.pdf]

bioRxiv preprint doi: https://doi.org/10.64898/2026.05.21.726063; this version posted May 26, 2026. The copyright holder for this preprint (which was not certified by peer review) is the author/funder, who has granted bioRxiv a license to display the preprint in perpetuity. It is made available under a CC-BY 4.0 International license.

Table S1. qPCR primers used in this study

Target Gene	Species	Direction	Sequence (5’→3’)
Sox2	Mouse	Forward	AAGAAAGGAGAGAAGTTTGGAGCC
Sox2	Mouse	Reverse	GAGATCTGGCGGAGAATAGTTGG
Oct3/4	Mouse	Forward	AGAACATGTGTAAGCTGCGG
Oct3/4	Mouse	Reverse	TCACACGGTTCTCAATGCTAG
Nanog	Mouse	Forward	AGGGTCTGCTACTGAGATGCTCTG
Nanog	Mouse	Reverse	CAACCACTGGTTTTTCTGCCACCG
SREBP1	Mouse	Forward	CGACTACATCCGCTTCTTGCAG
SREBP1	Mouse	Reverse	CCTCCATAGACACATCTGTGCC
SREBP2	Mouse	Forward	AGAAAGAGCGGTGGAGTCCTTG
SREBP2	Mouse	Reverse	GAACTGCTGGAGAATGGTGAGG
ACLY	Mouse	Forward	ACCCTTTCACTGGGGATCACA
ACLY	Mouse	Reverse	GACAGGGATCAGGATTTCCTTG
ACC1	Mouse	Forward	GATGAACCATCTCCGTTGGC
ACC1	Mouse	Reverse	GACCCAATTATGAATCGGGAGTG
ACC2	Mouse	Forward	CGCTCACCAACAGTAAGGTGG
ACC2	Mouse	Reverse	GCTTGGCAGGGAGTTCCTC
FASN	Mouse	Forward	GGCTCTATGGATTACCCAAGC
FASN	Mouse	Reverse	CCAGTGTTCGTTCCTCGGA
SCD1	Mouse	Forward	GCAAGCTCTACACCTGCCTCTT
SCD1	Mouse	Reverse	CGTGCCTTGTAAGTTCTGTGGC
CPT1a	Mouse	Forward	GGCATAAACGCAGAGCATTCCTG
CPT1a	Mouse	Reverse	CAGTGTCCATCCTCTGAGTAGC
GAPDH	Mouse	Forward	TCACCACCATGGAGAAGGC
GAPDH	Mouse	Reverse	GCTAAGCAGTTGGTGGTGCA
GAPDH	Human	Forward	GTCTCCTCTGACTTCAACAGCG
GAPDH	Human	Reverse	ACCACCCTGTTGCTGTAGCCAA
B2M	Mouse	Forward	ACAGTTCCACCCGCCTCACATT
B2M	Mouse	Reverse	TAGAAAGACCAGTCCTTGCTGAAG
H2-Db	Mouse	Forward	ACTCCAGCAGATGTCTGGCTGTGACT
H2-Db	Mouse	Reverse	GCCTTGTAATGCTCTGCAGCACCACT
H2-Kb	Mouse	Forward	GGCTCTCACACTATTCAGGTGATC
H2-Kb	Mouse	Reverse	CTCCAGGTAGGCCCTGAGT
HLA-A	Human	Forward	AGATACACCTGCCATGTGCAGC

bioRxiv preprint doi: https://doi.org/10.64898/2026.05.21.726063; this version posted May 26, 2026. The copyright holder for this preprint (which was not certified by peer review) is the author/funder, who has granted bioRxiv a license to display the preprint in perpetuity. It is made available under a CC-BY 4.0 International license.

**Target Gene**	**Species**	**Direction**	**Sequence (5'→3')**
HLA-A	Human	Reverse	GATCACAGCTCCAAGGAGAACC
HLA-B	Human	Forward	CTGCTGTGATGTGTAGGAGGAAG
HLA-B	Human	Reverse	GCTGTGAGAGACACATCAGAGC
HLA-C	Human	Forward	GGAGACACAGAAGTACAAGCGC
HLA-C	Human	Reverse	ACATCCTCTGGAGGGTGTGAGA

bioRxiv preprint doi: https://doi.org/10.64898/2026.05.21.726063; this version posted May 26, 2026. The copyright holder for this preprint (which was not certified by peer review) is the author/funder, who has granted bioRxiv a license to display the preprint in perpetuity. It is made available under a CC-BY 4.0 International license.

Table S2. shRNA constructs

Target	Name	Species	Sequence (5'→3')
B2m	B2M mir30-1	Mouse	GCCGAACATACTGAACTGCTA TAGTGAAGCCACAGATGTA TAGCAGTTCAGTATGTTCGGCT
B2m	B2M mir30-2	Mouse	CCAGTTTCTAATATGCTATAC TAGTGAAGCCACAGATGTA GTATAGCATATTAGAAACTGGA
B2m	B2M mir30-3	Mouse	TAAAGTAGAGATGTCAGATAT TAGTGAAGCCACAGATGTA ATATCTGACATCTCTACTTTAG

bioRxiv preprint doi: https://doi.org/10.64898/2026.05.21.726063; this version posted May 26, 2026. The copyright holder for this preprint (which was not certified by peer review) is the author/funder, who has granted bioRxiv a license to display the preprint in perpetuity. It is made available under a CC-BY 4.0 International license.

Table S3. CRISPR guides

Target	Species	Type	Sequence (5’→3’)
FASN	Mouse	sgRNA	GAGGAACGAACACTGGCGTC
FASN	Mouse	crRNA	/AltR1/rGrA rGrGrA rArCrG rArArC rArCrU rGrGrC rGrUrC rGrUrU rUrUrA rGrArG rCrUrA rUrGrC rU/AltR2/
FASN	Human	sgRNA	CGGCCGACTGGTACAACGAG
EGFR	Human	sgRNA	CCTTGCACGTGGCTTCGTCT

bioRxiv preprint doi: https://doi.org/10.64898/2026.05.21.726063; this version posted May 26, 2026. The copyright holder for this preprint (which was not certified by peer review) is the author/funder, who has granted bioRxiv a license to display the preprint in perpetuity. It is made available under a CC-BY 4.0 International license.

Table S4. Reagents and laboratory supplies used in this study

Product Name	Vendor	Catalog Number
DMEM (Dulbecco's Modified Eagle's Medium)	Corning	MT10027CV
DMEM	Corning	10-013-CV
Advanced RPMI 1640	Thermo Fisher Scientific	12633020
HEPES (1 M)	Corning	25-060-CI
Fetal Bovine Serum (FBS)	Corning	35-010-CV
Fetal Bovine Serum, heat-inactivated	Thermo Fisher Scientific	10082147
FBS, lipid-depleted	Captivate	FBS162LD
PBS (Phosphate Buffered Saline)	Corning	21-040-CV
HBSS	Thermo Fisher Scientific	14175-103
Trypsin-EDTA (0.25%)	Thermo Fisher Scientific	45000-664
TrypLE Express Enzyme	Thermo Fisher Scientific	12604021
Penicillin-Streptomycin	Thermo Fisher Scientific	45000-652
CellTiter-Glo Luminescent Assay	Promega	G7571
CellTiter 96 AQueous One Solution (MTS)	Promega	G358A
Neon Transfection System Kit (10 µL)	Thermo Fisher Scientific	MPK1096
Lipofectamine 3000	Thermo Fisher Scientific	L3000001
Opti-MEM Reduced Serum Medium	Thermo Fisher Scientific	31985062
Matrigel Basement Membrane Matrix	Corning	356231
Transwell Inserts (polycarbonate)	Corning	CLS3422
96-well Cell Culture Plates	VWR	29442-058
PCR Plate Sealing Film (Microseal B)	Bio-Rad	MSB1001
96-well PCR Plates (Low Profile)	Bio-Rad	MSP9601
Cell Extraction Buffer	Thermo Fisher Scientific	FNN0011
NP-40 Detergent	Thermo Fisher Scientific	85124
Tween 20 (10%)	Bio-Rad	161-0781
SDS (20%)	Thermo Fisher Scientific	AM9820
Tris Buffer (1 M, pH 7.4)	Thermo Fisher Scientific	J60202.K2
NaCl (5 M, RNase-free)	Thermo Fisher Scientific	AM9759
EDTA (0.5 M, pH 8.0)	Thermo Fisher Scientific	15575020
Formaldehyde Solution	Sigma-Aldrich	F8775-500
Neutral Buffered Formalin (10%)	Sigma-Aldrich	HT5012-1CS
Bovine Serum Albumin (BSA)	Sigma-Aldrich	A8412-100ML

bioRxiv preprint doi: https://doi.org/10.64898/2026.05.21.726063; this version posted May 26, 2026. The copyright holder for this preprint (which was not certified by peer review) is the author/funder, who has granted bioRxiv a license to display the preprint in perpetuity. It is made available under a CC-BY 4.0 International license.

Product Name	Vendor	Catalog Number
BSA, fatty acid-free	Sigma-Aldrich	126609-10GM
BSA	Sigma-Aldrich	A8806-5G
Calcium Chloride (2 M)	Fisher Scientific	50-751-7642
ACK Lysing Buffer	Quality Biological	118-156-101
RBC Lysis Buffer (1X)	Thermo Fisher Scientific	00-4333-57
Fix/Lyse Solution (10X)	Thermo Fisher Scientific	00-5333-54
Collagenase/Hyaluronidase	Stemcell Technologies	07912
DNase I (RNase-free)	Thermo Fisher Scientific	EN0521
Deoxyribonuclease I	Worthington	LS006333
Cell Staining Buffer	BioLegend	420201
Bottle-top Vacuum Filter System	Corning	CLS430769
Doxycycline hyclate	Millipore Sigma	D5207
Teklad doxycycline diet 7012, irradiated	Envigo	TD.08434
Pierce™ NEM (N-ethylmaleimide)	Thermo Fisher Scientific	23030
Biotin-HDPD	Abcam	ab145614
Streptavidin agarose	Sigma-Aldrich	S1638-1ML
Pierce™ IgG Elution Buffer	Thermo Fisher Scientific	21004
Pierce™ Protein A/G Magnetic Beads	Thermo Fisher Scientific	88802
Cholesteryl hemisuccinate	MedChemExpress	HY-W010800
N-Dodecyl-β-D-maltoside	MedChemExpress	HY-128974
Anti-DYKDDDDK tag Affinity Beads	Abcam	ab270704
3xFLAG™ Peptide	Sigma-Aldrich	F4799
Hydroxylamine	Sigma-Aldrich	438227
Blasticidin	InvivoGen	ant-bl-05
Puromycin (10 mg/ml solution)	InvivoGen	ant-pr-1
G418 (solution)	InvivoGen	ant-gn-5
chloroquine	Cayman Chemical	14194
Denifanstat (TVB-2640)	MedChemExpress	HY-112829
TVB 3166	MedChemExpress	HY-120394
PEG300 (Synonyms: Polyethylene glycol 300)	MedChemExpress	HY-Y0873
Tween 80 (Synonyms: Polysorbate 80)	MedChemExpress	HY-Y1891
Rapamycin	MedChemExpress	HY-10219
Torin	MedChemExpress	HY-13003
Dimethyl sulfoxide (Synonyms: DMSO)	MedChemExpress	HY-Y0320C

bioRxiv preprint doi: https://doi.org/10.64898/2026.05.21.726063; this version posted May 26, 2026. The copyright holder for this preprint (which was not certified by peer review) is the author/funder, who has granted bioRxiv a license to display the preprint in perpetuity. It is made available under a CC-BY 4.0 International license.

Product Name	Vendor	Catalog Number
Pierce™ BCA Protein Assay Kit	Thermo Fisher Scientific	23227

bioRxiv preprint doi: https://doi.org/10.64898/2026.05.21.726063; this version posted May 26, 2026. The copyright holder for this preprint (which was not certified by peer review) is the author/funder, who has granted bioRxiv a license to display the preprint in perpetuity. It is made available under a CC-BY 4.0 International license.

Table S5. Flow Cytometry Reagents

Target / Marker	Fluorophore	Product Name	Species	Vendor	Catalog #	Dilution
**General Reagents & Buffers**						
RBC Lysis	—	1X RBC Lysis Buffer	N/A	Thermo Fisher Scientific	00-4333-57	N/A
Staining Buffer	—	Cell Staining Buffer	N/A	Fisher Scientific	NC9742503	N/A
Flow Buffer	—	Flow Cytometry Staining Buffer	N/A	Thermo Fisher	00-4222-26	N/A
Fix/Perm	—	Fixation/Permeabilization Concentrate	N/A	Thermo Fisher	00-5123-43	1:10
Fix/Perm	—	Fixation/Permeabilization Diluent	N/A	Thermo Fisher	00-5223-56	N/A
Permeabilization	—	Permeabilization Buffer (10X)	N/A	Thermo Fisher	00-8333-56	1:10
Fc Block	—	Human BD Fc Block™	human	BD Biosciences	564220	1:100
Fc Block	—	TruStain FcX™ PLUS (CD16/32)	mouse	BioLegend	156604	1:100
**Viability / Apoptosis**						
Live/Dead	7-AAD	Viability Staining Solution	N/A	BioLegend	420404	1:100
Live/Dead	Zombie UV	Zombie UV	N/A	BioLegend	423108	1:100
DNA	DAPI	DAPI	N/A	Thermo Fisher	D1306	0.2 µg/mL
PI	—	Propidium Iodide Solution	N/A	BioLegend	421301	1:100
PI Kit	—	Propidium Iodide Kit	N/A	Abcam	ab139418	N/A
**T Cell Markers**						
CD3	BUV395	CD3 (17A2)	mouse	Thermo Fisher	363-0032-82	1:100
CD3	RB744	Rat Anti-Mouse CD3	mouse	BD Biosciences	570561	1:100
CD4	BUV737	Rat Anti-Mouse CD4	mouse	BD Horizon	612844	1:100

bioRxiv preprint doi: https://doi.org/10.64898/2026.05.21.726063; this version posted May 26, 2026. The copyright holder for this preprint (which was not certified by peer review) is the author/funder, who has granted bioRxiv a license to display the preprint in perpetuity. It is made available under a CC-BY 4.0 International license.

Target / Marker	Fluorophore	Product Name	Species	Vendor	Catalog #	Dilution
CD8a	AF488	CD8a	mouse	BioLegend	100723	1:100
CD8a	BV785	CD8a	mouse	BioLegend	100750	1:100
CD25	BUV737	CD25 (PC61.5)	mouse	Thermo Fisher	367-0251-82	1:100
CD25	BV750	CD25	mouse	BioLegend	102077	1:100
CD69	AF700	CD69	mouse	BioLegend	104539	1:100
CD69	BUV661	CD69	mouse	BD Biosciences	741478	1:100
CD62L	BV785	CD62L	mouse	BioLegend	104440	1:100
CD62L	PE-CF594	CD62L	mouse	BD Biosciences	562404	1:100
CD62L	RB744	CD62L	mouse	BD Biosciences	570965	1:100
CD44	AF488	CD44	mouse/ human	BioLegend	103016	1:100
CD44	AF700	CD44	mouse	BioLegend	156010	1:100
CD44	BV711	CD44	mouse/ human	BioLegend	103057	1:100
**Checkpoint / Exhaustion Markers**						
PD-1	BV711	CD279 (PD-1)	mouse	BioLegend	135231	1:100
PD-1	BUV395	PD-1 (J43)	mouse	Thermo Fisher	363-9985-82	1:100
PD-1	PE/Dazzle 594	PD-1	mouse	BioLegend	135228	1:100
PD-1	RB705	PD-1	mouse	BD Biosciences	570566	1:100
PD-L1	BV711	CD274 (PD-L1)	mouse	BioLegend	124319	1:100
PD-L1	BUV737	CD274 (MIH5)	mouse	Thermo Fisher	367-5982-82	1:100
LAG-3	BV711	CD223 (LAG-3)	mouse	BioLegend	125243	1:100
LAG-3	BUV737	CD223	mouse	Thermo Fisher	367-2239-42	1:100
Tim-3	BV785	CD366 (Tim-3)	mouse	BioLegend	119725	1:100

bioRxiv preprint doi: https://doi.org/10.64898/2026.05.21.726063; this version posted May 26, 2026. The copyright holder for this preprint (which was not certified by peer review) is the author/funder, who has granted bioRxiv a license to display the preprint in perpetuity. It is made available under a CC-BY 4.0 International license.

Target / Marker	Fluorophore	Product Name	Species	Vendor	Catalog #	Dilution
**Myeloid / Innate Markers**						
CD11b	BV605	CD11b	mouse/ human	BioLegend	101257	1:100
CD11c	PerCP-Cy5.5	CD11c	mouse	BioLegend	117328	1:100
Ly-6C	BUV563	Ly-6C	mouse	BD Biosciences	755198	1:100
Ly-6C	RB545	Ly-6C	mouse	BD Biosciences	569288	1:100
Ly-6G	PerCP-Fire 806	Ly-6G	mouse	BioLegend	127684	1:100
F4/80	RB780	F4/80	mouse	BD Biosciences	569223	1:100
CD206	AF700	CD206	mouse	BioLegend	141734	1:100
CD206	BV711	CD206	mouse	BioLegend	141727	1:100
**B / NK Cell Markers**						
B220	BV711	CD45R (B220)	mouse	BioLegend	103255	1:100
NK1.1	BUV395	NK1.1	mouse	BD Horizon	564144	1:100
NK1.1	BUV395	NK1.1 (PK136)	mouse	Thermo Fisher	363-5941-82	1:100
**Antigen Presentation / MHC**						
HLA-ABC	AF647	HLA-A,B,C	human	BioLegend	114612 / 311414	1:100
HLA-ABC	PE	HLA-A,B,C	human	BioLegend	311406	1:100
HLA-ABC	BUV395	HLA-ABC	human	BD Biosciences	567859	1:100
MHC-I	AF647	H-2Kb/H-2Db	mouse	BioLegend	114612	1:100
MHC-I	BUV395	H-2Kb/H-2Db	mouse	BD Biosciences	745596	1:100
SIINFEKL	AF647	H-2Kb/SIINFEKL	mouse	BD Biosciences	570011	1:100
SIINFEKL	BUV395	H-2Kb/SIINFEKL	mouse	BD Biosciences	570093	1:100
MHC-II	AF647	I-A/I-E	mouse	BioLegend	107618	1:100

bioRxiv preprint doi: https://doi.org/10.64898/2026.05.21.726063; this version posted May 26, 2026. The copyright holder for this preprint (which was not certified by peer review) is the author/funder, who has granted bioRxiv a license to display the preprint in perpetuity. It is made available under a CC-BY 4.0 International license.

Target / Marker	Fluorophore	Product Name	Species	Vendor	Catalog #	Dilution
MHC-II	Pacific Blue	I-A/I-E	mouse	BioLegend	107620	1:100
**Tumor / Epithelial Markers**						
EGFR	AF647	EGFR (528)	mouse	Santa Cruz	sc-120 AF647	1:100
EGFR	BV785	EGFR	human	BioLegend	352942	1:100
EpCAM	BV711	CD326 (EpCAM)	mouse	BioLegend	118233	1:100
CD31	AF647	CD31	mouse	BioLegend	102516	1:100
**Cytokines / Functional Markers**						
IFN-γ	BUV737	IFN-γ (XMG1.2)	mouse	Thermo Fisher	367-7311-82	1:100
TNF-α	BV785	TNF-α	mouse	BioLegend	506341	1:100
IL-2	BV421	IL-2	mouse	BioLegend	503826	1:100
CD107a	BV711	CD107a (LAMP-1)	mouse	BioLegend	121631	1:100
**Other Reagents**						
Brefeldin A	—	Brefeldin A (1000X)	N/A	BioLegend	420601	1:1000
Monensin	—	Monensin (1000X)	N/A	BioLegend	420701	1:1000
Ionomycin	—	Ionomycin (1000X)	N/A	MedChemExpress	HY-13434	1:1000
PMA	—	Phorbol 12-myristate 13-acetate	N/A	MedChemExpress	HY-18739	1:1000
Calibration	—	Quantum Alexa Fluor 647 MESF	N/A	Bangs Labs	647	N/A
Beads	—	Simply Cellular anti-Mouse IgG	mouse	Bangs Labs	815	1:100
Beads	—	Simply Cellular anti-Rat IgG	N/A	Bangs Labs	817	N/A

bioRxiv preprint doi: https://doi.org/10.64898/2026.05.21.726063; this version posted May 26, 2026. The copyright holder for this preprint (which was not certified by peer review) is the author/funder, who has granted bioRxiv a license to display the preprint in perpetuity. It is made available under a CC-BY 4.0 International license.

Table S6. Western Blot Reagents and Antibodies

Target / Category	Product Name	Vendor	Catalog #	Dilution
**Gel Electrophoresis & Transfer Reagents**				
Gel	4–20% Mini-PROTEAN® TGX™ Precast Gel (15-well)	Bio-Rad	4561096	N/A
Gel	4–20% Mini-PROTEAN® TGX™ Precast Gel (10-well)	Bio-Rad	4561094	N/A
Ladder	Precision Plus Protein™ Kaleidoscope™ Standards	Bio-Rad	1610395	N/A
Membrane	Immun-Blot PVDF Membrane	Bio-Rad	1620177	N/A
Transfer	Foam Pads for Mini Trans-Blot® Cell	Bio-Rad	1703933	N/A
Transfer Buffer	10× Tris/Glycine Blot Buffer	Bio-Rad	1610771	N/A
Running Buffer	10× Tris/Glycine/SDS Buffer	Bio-Rad	1610772EDU	N/A
**Blocking & General Buffers**				
Blocking	Bovine Serum Albumin (BSA)	Sigma	A8806-5G	5%
Loading Buffer	Blue Loading Buffer Pack	Cell Signaling Technology	7722S	—
Wash Buffer	Tris Buffered Saline (TBS)	Santa Cruz	sc-362186	1×
Stripping	Restore™ Stripping Buffer	Thermo Fisher	PI21063	N/A
Solvent	Methanol	VWR	TS42395-0025	20%
Blotting	Blotting-Grade Blocker	Bio-rad	1706404	5%
**Detection Reagents**				
Substrate	Amersham ECL Prime Detection Reagent	GE Healthcare	RPN2232	N/A
Film	Development Folders	Thermo Fisher	T2258	N/A
**Secondary Antibodies (HRP)**				
Anti-Mouse IgG	Goat anti-Mouse IgG (H+L), HRP	Thermo Fisher	31430	1:10,000

bioRxiv preprint doi: https://doi.org/10.64898/2026.05.21.726063; this version posted May 26, 2026. The copyright holder for this preprint (which was not certified by peer review) is the author/funder, who has granted bioRxiv a license to display the preprint in perpetuity. It is made available under a CC-BY 4.0 International license.

Target / Category	Product Name	Vendor	Catalog #	Dilution
Anti-Mouse IgG	Anti-Mouse IgG, HRP-linked	Cell Signaling Technology	7076S	1:10,000
Anti-Rabbit IgG	Anti-Rabbit IgG, HRP-linked	Cell Signaling Technology	7074S	1:10,000
**Loading Controls**				
β-Actin	β-Actin (D6A8) Rabbit mAb	Cell Signaling Technology	8457S	1:1,000
GAPDH	GAPDH (14C10) Rabbit mAb	Cell Signaling Technology	2118S	1:1,000
β2M	β-2 Microglobulin (4H5L6)	Thermo Fisher	701250	1:1,000
**Lipid Metabolism / SREBP Axis**				
FASN	Fatty Acid Synthase Antibody	Cell Signaling Technology	3189S	1:1,000
FASN	Fatty Acid Synthase (C20G5) Rabbit mAb	Cell Signaling Technology	3180S	1:1,000
SREBP1	Anti-SREBP1	Abcam	ab28481	1:1,000
SREBP2	SREBP2 Polyclonal Antibody	Thermo Fisher	PA1-338	1:1,000
**Epithelial–Mesenchymal Transition (EMT)**				
E-Cadherin	E-Cadherin (24E10) Rabbit mAb	Cell Signaling Technology	3195S	1:1,000
Vimentin	Vimentin (D21H3) Rabbit mAb	Cell Signaling Technology	5741S	1:1,000
**EGFR Signaling**				
EGFR (Total)	EGFR (D38B1) Rabbit mAb	Cell Signaling Technology	4267S	1:1,000
EGFR (Total)	EGFR Antibody (A-10)	Santa Cruz	sc-373746	1:1,000
EGFR (pY1068)	Phospho-EGFR (Tyr1068) (D7A5)	Cell Signaling Technology	3777S	1:1,000
EGFR (pY1068)	Phospho-EGFR (Y1068)	Abcam	ab5644	1:1,000
**PI3K–AKT–mTOR Pathway**				
PI3K p85 (Total)	PI3K p85 Antibody	Cell Signaling Technology	4292S	1:1,000
PI3K p85 (pTyr458)	Phospho-PI3K p85/p55	Cell Signaling Technology	17366S	1:1,000

bioRxiv preprint doi: https://doi.org/10.64898/2026.05.21.726063; this version posted May 26, 2026. The copyright holder for this preprint (which was not certified by peer review) is the author/funder, who has granted bioRxiv a license to display the preprint in perpetuity. It is made available under a CC-BY 4.0 International license.

Target / Category	Product Name	Vendor	Catalog #	Dilution
Akt (Total)	Akt Antibody	Cell Signaling Technology	9272S	1:1,000
Akt (pSer473)	Phospho-Akt (Ser473)	Cell Signaling Technology	9271S	1:1,000
mTOR (Total)	mTOR (7C10) Rabbit mAb	Cell Signaling Technology	2983S	1:1,000
mTOR (pSer2448)	Phospho-mTOR (Ser2448)	Cell Signaling Technology	5536S	1:1,000
p70 S6K (Total)	p70 S6 Kinase	Cell Signaling Technology	9202S	1:1,000
p70 S6K (pThr389)	Phospho-p70 S6 Kinase	Cell Signaling Technology	9205S	1:1,000
**MAPK Signaling**				
ERK1/2 (Total)	p44/42 MAPK (Erk1/2)	Cell Signaling Technology	9102S	1:1,000
ERK1/2 (pThr202/Tyr204)	Phospho-ERK1/2	Cell Signaling Technology	9101S	1:1,000
p38 (Total)	p38 MAPK	Cell Signaling Technology	9212S	1:1,000
p38 (pThr180/Tyr182)	Phospho-p38 MAPK	Cell Signaling Technology	9211S	1:1,000
**Energy Sensing**				
AMPKα (Total)	AMPKα Antibody	Cell Signaling Technology	2532S	1:1,000
AMPKα (pThr172)	Phospho-AMPKα	Cell Signaling Technology	2535S	1:1,000
**Tags / Miscellaneous**				
FLAG tag	Anti-DDDDK tag	Abcam	ab205606	1:1,000

bioRxiv preprint doi: https://doi.org/10.64898/2026.05.21.726063; this version posted May 26, 2026. The copyright holder for this preprint (which was not certified by peer review) is the author/funder, who has granted bioRxiv a license to display the preprint in perpetuity. It is made available under a CC-BY 4.0 International license.

Table S7. DNA Cloning and Molecular Biology Reagents

Category	Product Name	Vendor	Catalog #
**Plasmid Preparation & DNA Purification**			
Endotoxin-free plasmid prep	NucleoBond Xtra Midi Plus EF	MACHEREY-NAGEL	740422-5
Plasmid miniprep	Monarch® Plasmid Miniprep Kit	NEB	T1010S
PCR cleanup	Monarch® PCR & DNA Cleanup Kit	NEB	T1030S
Gel extraction	Monarch® DNA Gel Extraction Kit	NEB	T1020S
RNA isolation	RNeasy Plus Mini Kit	Qiagen	74134
DNase treatment	DNase I	Worthington	LS006333
**PCR Amplification & Mutagenesis**			
High-fidelity PCR	Q5® Hot Start High-Fidelity Master Mix	NEB	M0494S
High-fidelity PCR	iProof HF Master Mix	Bio-Rad	1725310
Site-directed mutagenesis	Q5® Site-Directed Mutagenesis Kit	NEB	E0552S
Additive	DMSO	VWR	97063-136
**DNA Assembly & Ligation**			
DNA assembly	NEBuilder® HiFi DNA Assembly Kit	NEB	E5520S
Ligation kit	Quick Ligation™ Kit	NEB	M2200S
DNA ligase	T4 DNA Ligase	NEB	M0202S
Dephosphorylation	Quick CIP	NEB	M0525S
Phosphorylation	T4 Polynucleotide Kinase	NEB	M0201S
**Restriction Enzymes**			
Type IIS enzyme	BsmBI-v2	NEB	R0739S
Restriction enzyme	SpeI-HF	NEB	R3133S
Restriction enzyme	XbaI	NEB	R0145S
**Bacterial Transformation & Culture**			
Competent cells	NEB® 5-alpha Competent *E. coli*	NEB	C2987H

bioRxiv preprint doi: https://doi.org/10.64898/2026.05.21.726063; this version posted May 26, 2026. The copyright holder for this preprint (which was not certified by peer review) is the author/funder, who has granted bioRxiv a license to display the preprint in perpetuity. It is made available under a CC-BY 4.0 International license.

Category	Product Name	Vendor	Catalog #
Competent cells	NEB® Stable Competent *E. coli*	NEB	C3040H
Competent cells	One Shot™ Stbl3™ *E. coli*	Thermo Fisher	C737303
Selection antibiotic	Ampicillin sodium salt	Thermo Fisher	611770250
Selection antibiotic	Chloramphenicol	MilliporeSigma	C0378-25G
**Buffers & Core Reagents**			
Water	Nuclease-Free Water	Invitrogen	AM9937
Buffer	Tris (1.0 M, pH 7.4)	Thermo Fisher	J60202.K2
Salt	NaCl (5 M, RNase-free)	Thermo Fisher	AM9759
Detergent	NP-40 Surfact-Amps™	Thermo Fisher	85124
Detergent	SDS (20% solution)	Thermo Fisher	AM9820
**cDNA Synthesis & qPCR**			
Reverse transcription	qScript cDNA SuperMix	Quanta Biosciences	95048-500
qPCR master mix	SsoAdvanced™ SYBR® Green Supermix	Bio-Rad	1725274
DNA stain	SYBR™ Safe DNA Gel Stain	Thermo Fisher	S33102
**General Laboratory Reagents**			
Solvent	2-Propanol	VWR	BT216010-4X1L
Solvent	Ethanol (absolute)	MilliporeSigma	E7023-6X500ML

bioRxiv preprint doi: https://doi.org/10.64898/2026.05.21.726063; this version posted May 26, 2026. The copyright holder for this preprint (which was not certified by peer review) is the author/funder, who has granted bioRxiv a license to display the preprint in perpetuity. It is made available under a CC-BY 4.0 International license.

Table S8. Organoid Media Reagents

**Reagent / Component**	**Supplier**	**Catalog number**
Advanced DMEM/F12	Invitrogen	12634-034
GlutaMAX (100×)	Invitrogen	12634-034
HEPES	Invitrogen	15630-056
Penicillin/Streptomycin	Invitrogen	15140-122
Primocin	InvivoGen	ant-pm-1
B27 Supplement	Gibco	17504-044
N-Acetylcysteine	Sigma-Aldrich	A9165-5G
Nicotinamide	Sigma-Aldrich	N0636
R-Spondin 1 conditioned medium	In-house	—
Recombinant human R-Spondin 3	R&D Systems	3500-RS/CF
Recombinant human Neuregulin-1	PeproTech	100-03
Recombinant human FGF7	PeproTech	100-19
Recombinant human FGF10	PeproTech	100-26
Recombinant human EGF	PeproTech	AF-100-15
Recombinant human Noggin	PeproTech	120-10C
A83-01	Tocris	2939
Y-27632	AbMole	Y-27632
SB202190	Sigma-Aldrich	S7067
β-Estradiol	Sigma-Aldrich	E2257
Forskolin	Sigma-Aldrich	F6886
Nutlin-3a	Merck	SML0580
Hydrocortisone	Sigma-Aldrich	H0888
Wnt surrogate	U-Protein Express BV	N001
